# Interactions among mTORC, AMPK and SIRT: a computational model for cell energy balance and metabolism

**DOI:** 10.1186/s12964-021-00706-1

**Published:** 2021-05-20

**Authors:** Mehrshad Sadria, Anita T. Layton

**Affiliations:** 1 Department of Applied Mathematics, University of Waterloo, Waterloo, ON Canada; 2 Department of Biology, Cheriton School of Computer Science, and School of Pharmacy, University of Waterloo, Waterloo, ON Canada

**Keywords:** Ageing, Autophagy, Growth factor signaling, Metabolism, Proliferation, MTOR, NAD+, Longevity, Sirtuins, System biology

## Abstract

**Background:**

Cells adapt their metabolism and activities in response to signals from their surroundings, and this ability is essential for their survival in the face of perturbations. In tissues a deficit of these mechanisms is commonly associated with cellular aging and diseases, such as cardiovascular disease, cancer, immune system decline, and neurological pathologies. Several proteins have been identified as being able to respond directly to energy, nutrient, and growth factor levels and stress stimuli in order to mediate adaptations in the cell. In particular, mTOR, AMPK, and sirtuins are known to play an essential role in the management of metabolic stress and energy balance in mammals.

**Methods:**

To understand the complex interactions of these signalling pathways and environmental signals, and how those interactions may impact lifespan and health-span, we have developed a computational model of metabolic signalling pathways. Specifically, the model includes (i) the insulin/IGF-1 pathway, which couples energy and nutrient abundance to the execution of cell growth and division, (ii) mTORC1 and the amino acid sensors such as sestrin, (iii) the Preiss-Handler and salvage pathways, which regulate the metabolism of NAD+ and the NAD+ -consuming factor SIRT1, (iv) the energy sensor AMPK, and (v) transcription factors FOXO and PGC-1α.

**Results:**

The model simulates the interactions among key regulators such as AKT, mTORC1, AMPK, NAD+ , and SIRT, and predicts their dynamics. Key findings include the clinically important role of PRAS40 and diet in mTORC1 inhibition, and a potential link between SIRT1-activating compounds and premature autophagy. Moreover, the model captures the exquisite interactions of leucine, sestrin2, and arginine, and the resulting signal to the mTORC1 pathway. These results can be leveraged in the development of novel treatment of cancers and other diseases.

**Conclusions:**

This study presents a state-of-the-art computational model for investigating the interactions among signaling pathways and environmental stimuli in growth, ageing, metabolism, and diseases. The model can be used as an essential component to simulate gene manipulation, therapies (e.g., rapamycin and wortmannin), calorie restrictions, and chronic stress, and assess their functional implications on longevity and ageing‐related diseases.

Video Abstract

**Supplementary Information:**

The online version contains supplementary material available at 10.1186/s12964-021-00706-1.

## Background

Across the board, single- and multi-cellular organisms exhibit the ability to sense nutrient fluctuations in their surrounding and to adjust their consumption accordingly. This ability allows an organism to undergo cellular proliferate and grow when nutrients are plentiful, and to conserve resources and survive when deprived of nutrients. This adaptation occurs at the cellular level, attained via a delicate balance between energy-consuming anabolic processes and energy-producing catabolic processes. This balancing act involves a number of signalling pathways, most notably the mechanistic target of rapamycin (mTOR) pathway. mTOR is a highly conserved serine/threonine protein kinase with two distinct complexes, mTORC1 and mTORC2. mTOR controls cell growth, proliferation, motility and survival, protein and lipid synthesis, glucose metabolism, mitochondrial function and transcription, in response to nutrient and hormonal signals [1].

mTORC1 is regulated by a variety of signals, including growth factors, glucose, and amino acids (AAs). In particular, the mTOR pathway is coupled to the insulin/IGF-1 (Insulin-like Growth Factor 1) signaling pathway. Insulin/IGF-1 activates mTORC1 by AKT (a.k.a. protein kinase B), while mTORC1 inhibits insulin/IGF-1 through S6K by inhibiting insulin receptor substrate (IRS) [2]. As such, the presence of nutrients and growth factors activates mTORC1, which promotes cell growth by stimulating a series of anabolic processes that include protein synthesis, and by inhibiting catabolic processes such as autophagy. Conversely, calorie restriction inhibits mTORC1, favoring catabolic processes, which produce sufficient energy and nutrients needed for survival. Inhibition of this nutrient response pathway is known to extend lifespan in model organism and ameliorates age-related pathologies [2].

Besides mTORC1, another key player in the ageing process is the sirtuin family, which consists of highly conserved protein deacetylases found nearly in all organisms studied. In mammals, seven silent information regulator (SIRT) proteins (SIRT1-7) exist, with SIRT1 being the most extensively studied in the context of ageing and is known as one of the main mediators in calorie restriction [3]. SIRT1 senses changes in intracellular nicotinamide adenine dinucleotide (NAD+) levels, and uses this information to adapt the cellular energy output. SIRT1 also enhances DNA repair, cell survival, mitochondrial function and reduces ageing inflammatory/immune responses [4].

Recent high-throughput genomic and proteomic technologies have generated a wealth of ageing-related data. Nonetheless, some of the molecular mechanisms that mediate key metabolism and ageing effects have yet to be elucidated. The difficulty lies in the complexity of the signaling pathways: Not only are a large number of genes and proteins involved, many with competing roles, but their interactions are complex and often incompletely characterized. Indeed, due to the multiple feedback loops and regulatory mechanisms, it is challenging to understand the biological consequences of gene and protein-expression changes. A promising methodology for interpreting data and untangling the interactions among signaling pathways is computational biology. One such approach is to describe regulatory interactions using ordinary differential equations (ODEs), which relate changes in the expressions of model variables to other quantities. The insulin/IGF-1 pathway has been the subject of modeling and analysis by a series of previous studies [5–12].

Unlike the insulin/IGF-1 and mTOR pathways, theoretical effort in modeling the arguably equally important regulators NAD+ and SIRT1 is limited (an exception is [13]). Thus, the goal of this study is to develop a state-of-the-art computational model that couples these and other critical signaling pathways in growth, ageing, metabolism, and disease in mammals. To achieve that goal, we present a comprehensive model that includes (i) the insulin/IGF-1 pathway, which couples energy and nutrient abundance to the execution of cell growth and division, (ii) mTORC1 and the AA sensors, (iii) the Preiss-Handler and salvage pathways, which regulate the metabolism of NAD+ and the NAD+ -consuming factor SIRT1, (iv) the energy sensor adenosine monophosphate-activated protein kinase (AMPK), and (v) transcription factors forkhead box O (FOXO) and peroxisome proliferator-activated receptor gamma coactivator 1-α (PGC-1α), the overexpression or mutation of which affects lifespan and health-span [14]. We apply the model to investigate the synergy among regulators of nutrients, energy, metabolism, and autophagy, and to identify novel therapeutic targets. The model can be used to aid in the interpretation of genomic and proteomic data, and to provide an integrated understanding of the mechanisms that lead the cell to senescence and how this process contributes to metabolic disorders and ageing-related diseases.

## Methods

**Fig. 1:**
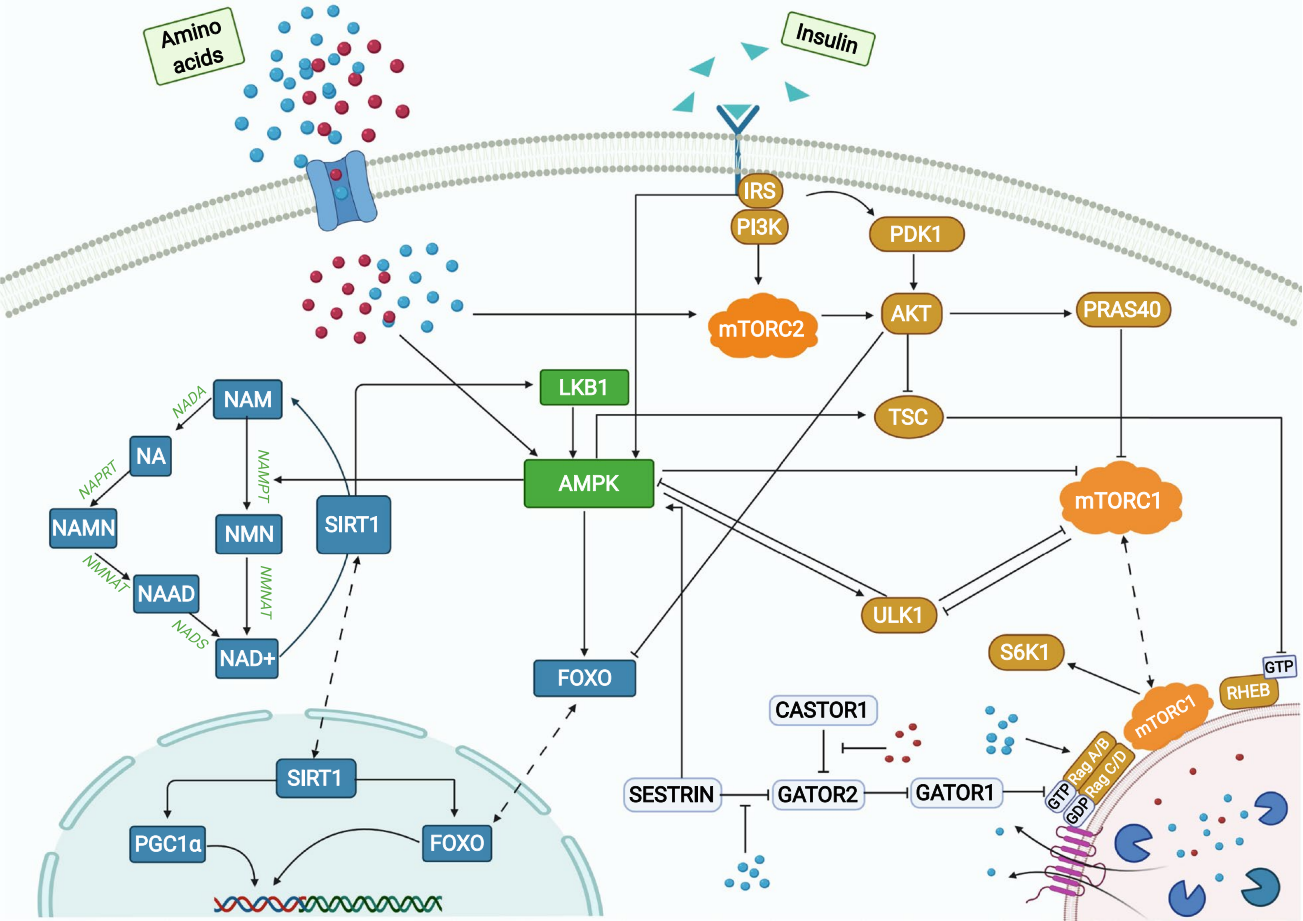
Pathway representation of metabolism model. Three distinct areas in the cell are represented: cytoplasm, lysosome, and nucleus. Key components in the insulin/IGF-1/mTOR signaling pathway are represented in brown, components in the Preiss-Handler and salvage pathways are in blue, and AMPK, which couples the two major pathways, is in green. Some proteins may be activated by AAs, such as leucine (blue circles) and arginine (red circles), and insulin (green triangles). AA sensors (e.g., sestrin2) are represented in white rounded rectangles. Solid arrows represent biochemical reactions, dashed arrows represent protein translocation

The present model is based primarily on the mouse C2C12 myoblast cell line. Main model components include the insulin/IGF-1 or mTOR signaling pathway [15], the Preiss-Handler and salvage pathways [16], energy sensor AMPK, and transcription factor FOXO and PGC-1α. The dynamics of the signaling pathways is modeled as a large system of coupled ODEs and algebraic equations. A schematic diagram that depicts key proteins and their interactions is shown in Fig. 1. A more comprehensive diagram can be found in Additional file 2: Fig. S1, where the activated and inactivated forms of a protein are represented separately, together with their individual interactions, to depict the pathways and protein interactions. The reactions and associated parameters are presented in Additional file 1: Table S1 and the Excel file. Model parameters are taken from published studies [11, 13, 17, 18] and fitted to experimental data [11, 17, 19, 20] using the interior point optimization method. The vast majority of the kinetic rates and data are from mice C2C12 myoblast cells [11, 13]. However, due to the size of the model and lack of enough experimental data, parameter identification is also supplemented using limited data from mouse kidney cells [17], rat cells [18], and human cells [19, 20]. The issue of cell-specific behaviors is addressed in the Discussion.

Within the insulin/IGF-1 signaling and mTOR pathways, insulin activates the insulin receptor (IR), which triggers the IRS and PI3K, resulting in the phosphorylation of PDK1 and mTORC2, respectively. mTORC2 phosphorylates AKT on the S473 and T308 residues, whereas PDK1 activates AKT. Actived AKT phosphorylates a variety of proteins, including FOXOs, TSC1_TSC2 and PRAS40. Phosphorylation of TSC1_TSC2 by AKT inactivates the TSC complex, thereby activating mTORC1 and resulting in a number of downstream effects. These include (i) inhibition of ULK1 by phosphorylating ULK1 on Ser 757; the ULK1 complex is a key contributor to the initiation of autophagy; (ii) phosphorylation of PRAS40, which attenuates the inhibition of mTORC1; (iii) phosphorylation of p70S6K on T229 and T389, which initiates a negative feedback loop via IRS [1].

An important novel aspect of our model is the explicit representation of the role of sestrin2 and leucine in the regulation of mTORC1. Sestrin2 deactivates mTORC1, via its effects on protein complexes GATOR1 and GATOR2. GATOR1 inhibits mTORC1, whereas GATOR2 inhibits GATOR1. Sestrin2 inhibits GATOR2, enhancing the activation of GATOR1, and eventually suppressing mTORC1. This process is modulated by leucine, which binds to sestrin2 and impedes its inhibitory effect on mTORC1 [21]. Thus, mTORC1 activation depends, among other factors, on the concentration of sestrin2 and leucine. To illustrate that dependence, we consider the mTORC1 activation rate:

1 $$\frac{d}{dt}\left[ {{\text{mTORC1\_pS2448}}} \right] = - k_{{{\text{TSC}}}} \left[ {{\text{mTORC1\_pS2448}}} \right]\left( {\left[ {TSC1\_TSC2} \right] + \left[ {TSC1\_TSC2\_pS1387} \right]} \right) + k_{{{\text{AA}}}} \left[ {{\text{mTORC1}}} \right]\left( {\frac{{V_{{{\text{max}}}} \left( {\left[ {\text{L}} \right] + c_{{{\text{LS}}}} } \right)}}{{\left[ {\text{S}} \right] + \left[ {\text{L}} \right] + c_{{{\text{LS}}}} }}} \right) - f_{1} \left( {{\text{[PRAS}}40]} \right) - f_{2} \left( {\text{[Act ULK1]}} \right)$$

where [L], [S], and c_LS_ denote leucine, sestrin2, and other AA (e.g., lysine) concentrations, respectively. Act ULK1 denotes the activated form of ULK1. The first term on the right describes the inhibition of mTORC1 by the TSC1_TSC2 complexes; the second term describes the effects of leucine and sestrin2 on mTORC1; the third and fourth terms ($f_{1}$ and $f_{2}$) describe the contributions from PRAS40 and activated ULK1, respectively. The expression for $f_{1}$ and $f_{2}$ can be found in the supporting material.

The model simulates the dynamics of another key player in metabolism, NAD+ , which is produced through the de novo, Preiss-Handler and salvage pathways. The major source of NAD+ in mammals is the salvage pathway, which recycles NAM produced by enzymes utilizing NAD+ [22]. The first step in the salvage pathway involves the rate-limiting enzyme NAMPT, which facilitates the conversion of NAM to NMN and whose activity is increased by AMPK, and the reduced expression and/or activity of which is associated with ageing and poor health [23]. The second step converts NMN to NAD+ via the NMNAT enzymatic reaction. NAD+ thus produced activates substrates including SIRT1 and is consumed in the process. Albeit less important for NAD+ biosynthesis in mammals, the Preiss-Handler pathway is also represented. The pathway begins with the conversion of NAM to NA, followed by the conversion to NAMN, catalyzed respectively by NADA and NAPRT. Like the salvage pathway, NMNAT in Preiss-Handler pathway also catalyzes the process of production of NAAD from NAMN. Finally, the reamidation of NAAD by NADS yields NAD+ .

Connecting the mTORC1 and NAD+ pathways is AMPK, a master regulator of cellular energy that is activated under starvation or hypoxia. AMPK can be stimulated by sestrin2, IRS, and liver kinase B1 (LKB1), which is deacetylated by SIRT1. Activated AMPK promotes autophagy by directly phosphorylating and activating ULK1 [24]. As such, there is a competition between mTORC1 and AMPK to phosphorylate different residues of ULK1 to decide cells fate. ULK1 in turn inhibits AMPK and mTORC1 in a negative feedback loop, whereas leucine activates them. The model also represents the stimulation by AMPK of TSC1_TSC2, the natural inhibitor of Rheb, which in turn decreases the activation of mTORC1.

We apply the model to simulate therapeutic treatments and different dietary conditions by changing selected model parameters:

- *Rapamycin*. Acute administration of rapamycin (e.g., 2 days following treatment) is simulated by decreasing total mTORC1 by 75% [25]. With chronic rapamycin administration (e.g., 5 weeks following treatment), mTORC2 is targeted in addition to mTORC1 [26]; thus, chronic administration is simulated by decreasing both mTORC1 and mTORC2 by 75%. These parameters were chosen based on findings reported in Ref. [25].
- *Glucose tolerance test*. Plasma glucose concentration is given by
  2 $$\frac{d\left[ G \right]}{{dt}} = k_{G,in} {-} k_{G,meta} \left[ G \right]\left[ {{\text{AKT\_pT308\_pS472}}} \right]$$

The cellular uptake rate of plasma glucose, the first step in cellular metabolism of glucose, is assumed to be proportional to the cellular concentration of AKT_pT308_pS472 [27]. In these simulations, the model is initialized at the fasting state with a low insulin level, glucose = 1, and $k_{G,in}$ = 0 which represents the absence of glucose intake; additionally, $k_{G,meta}$  = 7 × 10^–4^/min. At t = 40 min, the glucose tolerance test begins, whereby plasma glucose is elevated by setting $k_{G,in}$ to 0.115/min then linearly decreases to 0 in the 25 min following t = 40 min. Additionally, insulin is taken to be proportional to plasma glucose.

For a given set of parameters, a steady-state solution can be computed by integrating model equations for a sufficiently long time. For simulations that involve a change in model parameters, model variables are initialized to the steady-state solution corresponding to the parameter values at the initial time. Such initial conditions are realistic and avoid an abrupt change in solution at the initial time.

Sensitivity of model output *x* to a parameter *p* is given by the relative change in *x* with respect to a 1% relative change in *p*, i.e.,

3 $${\text{Sensitivity}} = \frac{{\left( {{\raise0.7ex\hbox{${x\left( {p + \Delta p} \right)}$} \!\mathord{\left/ {\vphantom {{x\left( {p + \Delta p} \right)} {x\left( p \right)}}}\right.\kern-\nulldelimiterspace} \!\lower0.7ex\hbox{${x\left( p \right)}$}}} \right) - 1}}{{{\raise0.7ex\hbox{${\Delta p}$} \!\mathord{\left/ {\vphantom {{\Delta p} p}}\right.\kern-\nulldelimiterspace} \!\lower0.7ex\hbox{$p$}}}}$$

where in our computations, $\Delta p = 0.01 p$. Other parameters are fixed and all model outputs are updated simultaneously when *p* is varied.

## Results

### Pharmacological suppression of mTORC1

Given the role of mTORC1 in metabolic diseases, there is great interest in developing drugs to suppress this protein complex. Rapamycin, a potent and selective inhibitor of mTORC1, has emerged as an FDA-approved immunosuppressant and anti-cancer agent. We conduct simulations to investigate the molecular mechanisms underlying the effects of rapamycin and its interactions with other mTORC1 inhibitors [28].

*How do nutritional levels affect the actions of mTORC1 inhibitors?* Because hyperactivation of mTORC1 disrupts cellular homeostasis, mTORC1 is regulated by a number of mechanisms, some of which are included in the present model. Both TSC1_TSC2 and its S1387-phosphorylated form deactivate mTORC1, as does the (dephosphorylated) proline-rich AKT substrate of 40 kDa (PRAS40). Compared to TSC1_TSC2, the regulation by PRAS40 of mTORC1 is less well studied; in fact, PRAS40’s effect on mTORC1 was not represented in a recent detailed mTORC pathway model [11]. Located at the crossroad of the insulin/IGF-1 pathway, PRAS40 is phosphorylated by growth factors or other stimuli, and in turn regulates the activation of these signaling pathways. PRAS40 plays an important role in metabolic disorders and multiple cancers, and is known to be an insulin-regulated inhibitor of mTORC1 [29]. To assess how that regulation is altered by nutritional levels and pharmacological interventions, we develop the first metabolism model that includes the actions of PRAS40. In these simulations, the model is initialized at the fasting state with a low insulin level (10% of baseline level). At t = 40 min, insulin level is returned to baseline and maintained at that level.

**Fig. 2:**
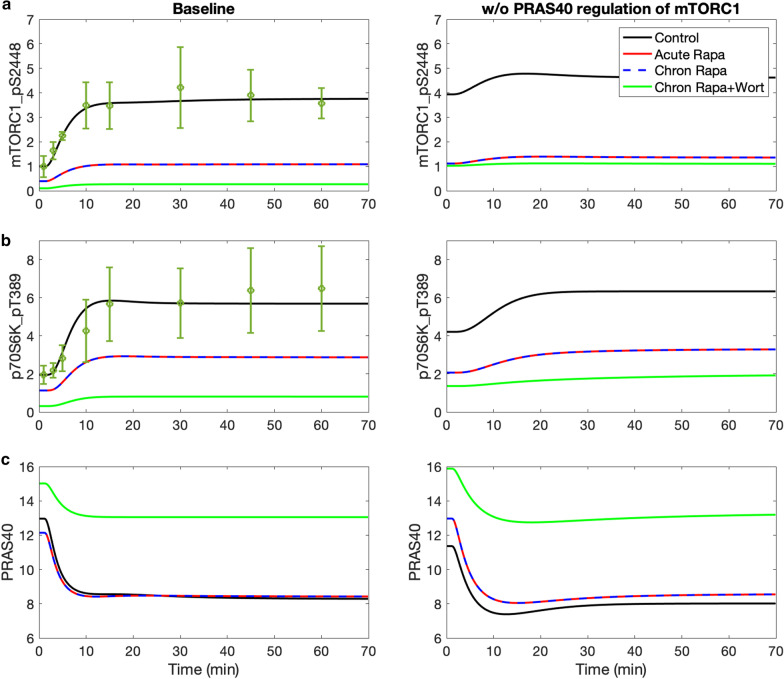
Effects of insulin, rapamycin, and wortmannin on key proteins and their interactions. Insulin was lowered to 10% of its baseline level for the initial 40 min of the simulation, and subsequently returned to baseline level. Simulations are conducted for control, acute and chronic administration of rapamycin, chronic administration of rapamycin with wortmannin. The inhibition of PRAS40 of mTORC1 is represented in the left panels but not the right ones. Included also are experimental time-course data for mTORC1_pS2448 and p70S6K_pT389 [11]; points and dotted error bars represent mean and SEM. Model predicts that PRAS40 substantially lowers mTORC1 level under low insulin conditions. That effect is the most prominent under chronic administration of rapamycin and wortmannin

The inhibitory effect of PRAS40 is contingent on the nutritional level. With sufficient insulin and no drug treatment (i.e., control; administration of rapamycin will be considered below), PRAS40 inhibition of mTORC1 has only a minor impact on mTORC1, lowering its steady-state phosphorylated level by 18% (compare Fig. 2a, left and right panels, black curves, t > 40 min). The effects of PRAS40 on other proteins are similarly minor (Fig. 2; Additional file 2: Figs. S2 and S3). In contrast, at low insulin level, PRAS40 inhibition of mTORC1 results in a substantially lower phosphorylated mTORC1 level (1.00 versus 3.94; compare Fig. 2a panels, black curves, t < 40 min). A lower insulin level decreases AKT_pT308_S473 (Additional file 2: Fig. S2b) and thus phosphorylated mTORC1; both changes reduce the phosphorylation rate of PRAS40 (Fig. 2c). The resulting higher unphosphorylated PRAS40 level reduces mTORC1 phosphorylation rate. The predicted profiles are consistent with experimental data in Ref. [11].

*What are the factors that affect the effectiveness of rapamycin, an mTORC1 inhibitor and anti-cancer agent?* Specifically, we simulate acute and chronic administration of rapamycin, and seek to answer the question: *Do rapamycin and PRAS40, both of which inhibit mTORC1, interact and produce nonlinear effects?* One might expect that together, rapamycin and PRAS40 may produce more than the sum of individual effects. This is because rapamycin lowers activated mTORC1 level and, as a result, decreases the phosphorylation of PRAS40 to PRAS40_pS183. Taken in isolation, that would increase dephosphorylated PRAS40 and further inactivate mTORC1. To assess the validity of that hypothesis, we simulate the administration of rapamycin with and without PRAS40 regulation. Model simulations predict that, following acute and chronic administration of rapamycin, mTORC1 level decreases by about 75% from its control level (Fig. 2a,b), which suggests limited super-additive effect arising from any interactions between rapamycin and PRAS40.

*Why doesn’t PRAS40 augment the inhibitory effect of rapamycin?* As noted above, the lower phosphorylated mTORC1 level, taken in isolation, would increase dephosphorylated PRAS40. However, through a feedback mechanism, the lower phosphorylated mTORC1 level also decreases p70S6K_pT389, increases phosphorylated IRS and phosphorylated PI3K_PDK1, and eventually, increases AKT_pT308 and AKT_pT308_pS473, both of which increase the phosphorylation of PRAS40 to PRAS40_pT246. The competing effects on PRAS40 phosphorylation result in negligible change in the unphosphorylated PRAS40 level.

Following chronic rapamycin administration, the lower phosphorylated mTORC2 level slows the phosphorylation of AKT to AKT_pS473, reducing both AKT_pS473 and the downstream AKT_pT308_pS473 (Fig. 2a,b). Taken in isolation, these effects, together with the reduced phosphorylated mTORC1, would slow the phosphorylation of PRAS40 and increase dephosphorylated PRAS40 level. But in a competing effect, the lower phosphorylated mTORC1 level increases AKT_pT308, which increases the phosphorylation of PRAS40 to PRAS40_pT246. These competing effects together yield negligible change in unphosphorylated PRAS40 level (Fig. 2c). Therefore, acute or chronic administration of rapamycin alone does not significantly alter PRAS40 levels.

*How can we augment the inhibition of mTORC1 by rapamycin, especially at high insulin level?* We hypothesize that this be achieved by pharmacological manipulations that elevate PRAS40. To identify an effective compound, we note that in addition to mTORC1, PRAS40 is also phosphorylated by AKT. This motivates us to consider inhibitors of PI3K_PDK1, which phosphorylate and activates AKT. We simulate such an inhibitor, wortmannin, by lowering total PI3K_PDK1 by 80% [30].

A noticeable effect can be observed in PRAS40 when wortmannin is combined with chronic rapamycin administration. The lower phosphorylated PI3K_PDK1 level decreases AKT_pT308 and AKT_pT308_pS473, slowing the phosphorylation of PRAS40 to PRAS40_pT246. Together with reduced phosphorylated mTORC1, this maneuver substantially increases unphosphorylated PRAS40 at baseline insulin by 55% (Fig. 2c, left). The elevated PRAS40 substantially suppresses mTORC1_pS2448 (Fig. 2a, left). Analogous effects are also obtained for MK-2206, an allosteric inhibitor of AKT.

*Optimizing rapamycin dosage to maintain insulin sensitivity while preserving mTORC1 inhibition.* Cells that exhibit insulin resistance produce an impaired response to insulin and fail to adequately metabolize glucose. Insulin resistance, which may be induced by excess nutrients, can be prevented by acute treatment with rapamycin. In contrast, the chronic administration of rapamycin may lead to insulin resistance via the inhibition of mTORC2, resulting in the attenuation of AKT_pT308_pS473, which is essential in the translocation of GLUT4 [31, 32] and the cellular entry and subsequence metabolism of glucose. In the next set of simulations, we explore the possibility that an optimal rapamycin dosage may be determined that attenuates its detrimental effect on insulin sensitivity while preserving its inhibition of mTORC1. To accomplish that goal, we simulate the glucose todlerance test (i.e., the model’s ability to metabolize glucose) under four conditions: control, acute rapamycin administration, chronic rapamycin administration, and chronic rapamycin and wortmannin administration (parameters as described in previous simulations). For chronic rapamycin, we considered mTORC2 inhibition at 75% (baseline), but also at 65%, 50%, and 25%, with mTORC1 inhibition fixed at 75%.

**Fig. 3:**
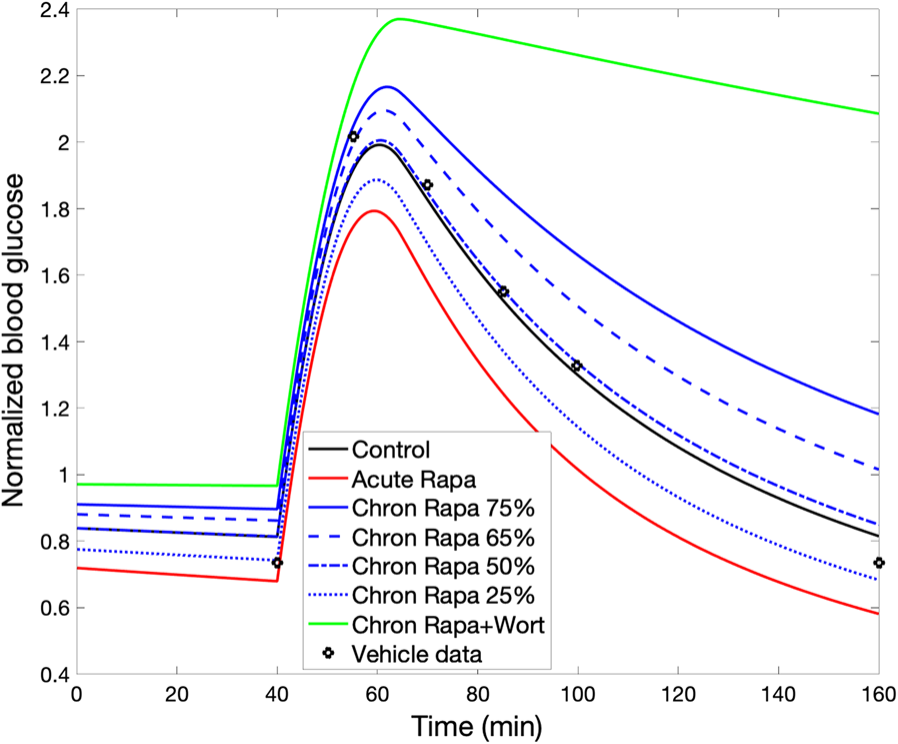
Effect of rapamycin on glucose tolerance. Glucose tolerance test begins at t = 40 min. Normalized plasma glucose profiles are obtained for acute rapamycin administration, and for chronic rapamycin administration at different dosages, with and without wortmannin. Vehicle data from [33]

The predicted plasma glucose time-course profiles are shown in Fig. 3, together with experiment data obtained for vehicle.[33] Acute rapamycin usage inhibits mTORC1 (but not mTORC2), which suppresses p70S6K_pT389, increases phosphorylated IRS, AKT_pT308 and AKT_pT308_pS473, thereby improving insulin sensitivity. In contrast, chronic rapamycin usage inhibits mTORC2 as well. That lowers AKT_pS473 and AKT_pT308_pS473, and, at sufficiently high dosage (> 50% mTORC2 inhibition), leads to impaired glucose tolerance. If wortmannin is added, AKT_pT308_pS473 is further suppressed, resulting in a sustained elevated plasma glucose level.

Findings in PC3 cells (a human prostate cancer cell line) suggest that differing rapamycin dosages may yield near maximal mTORC1 inhibition with a range of mTORC2 inhibition levels [25]. Model simulations suggest that lowering rapamycin-induced mTORC2 inhibition from 75% (baseline) to 50% restores insulin sensitivity to control level, consistent with intermittent administration of rapamycin [26, 34]. If it is possible to further reduce mTORC2 inhibition to 25% while preserving mTORC1 inhibition at 75%, one even achieves an improvement in insulin sensitivity, due to the beneficial effect of mTORC1 inhibition overriding the impairment arising from the (attenuated) mTORC2 inhibition.

## Amino acid sensors and mTORC1 regulation

As noted above, hyperactivation of the mTORC1 is implicated in the pathogenesis of cancer and other ageing-related diseases. mTORC1 promotes growth in response to the availability of nutrients, such as AAs, which drive mTORC1 to the lysosomal surface, its site of activation. Among the twenty classical L-amino acids, arginine and leucine are two essential AAs that potently stimulate the activity of mTORC1. However, aspects of the molecular mechanisms by which these specific AAs stimulate mTORC1 activity remain to be completely understood. Thus, in a set of simulations, we examine the roles of these AA sensors in mTORC1 regulation.

We first investigate the regulation of mTORC1 by leucine and its sensor, sestrin2. Sestrin2 and leucine exert opposite effect on mTORC1, with sestrin2 deactivating mTORC1, and leucine increasing mTORC1 activity by binding to sestrin2. To investigate the dependence of mTORC1 activation level on leucine and sestrin2, we consider the steady-state formulation of Eq. 1 (by setting the time-derivative to zero) and solve for [mTORC1_pS2448]. We obtain

4 $$\left[ {{\text{mTORC1\_pS2448}}} \right] = \frac{{k_{{{\text{AA}}}} {\text{mTORC1}}\left( {\frac{{V_{{{\text{max}}}} \left( {\left[ {\text{L}} \right] + c_{{{\text{LS}}}} } \right)}}{{\left[ {\text{S}} \right] + \left[ {\text{L}} \right] + c_{{{\text{LS}}}} }}} \right)}}{{k_{{{\text{TSC}}}} \left( {\left[ {TSC1\_TSC2} \right] + \left[ {TSC1\_TSC2\_pS1387} \right]} \right) + k_{{{\text{AA}}}} \frac{{V_{{{\text{max}}}} \left( {\left[ {\text{L}} \right] + c_{{{\text{LS}}}} } \right)}}{{\left[ {\text{S}} \right] + \left[ {\text{L}} \right] + c_{{{\text{LS}}}} }} - f_{1} \left( {\left[ {{\text{PRAS}}40} \right]} \right) - f_{2} \left( {\left[ {{\text{Act ULK}}1} \right]} \right)}}$$

**Fig. 4:**
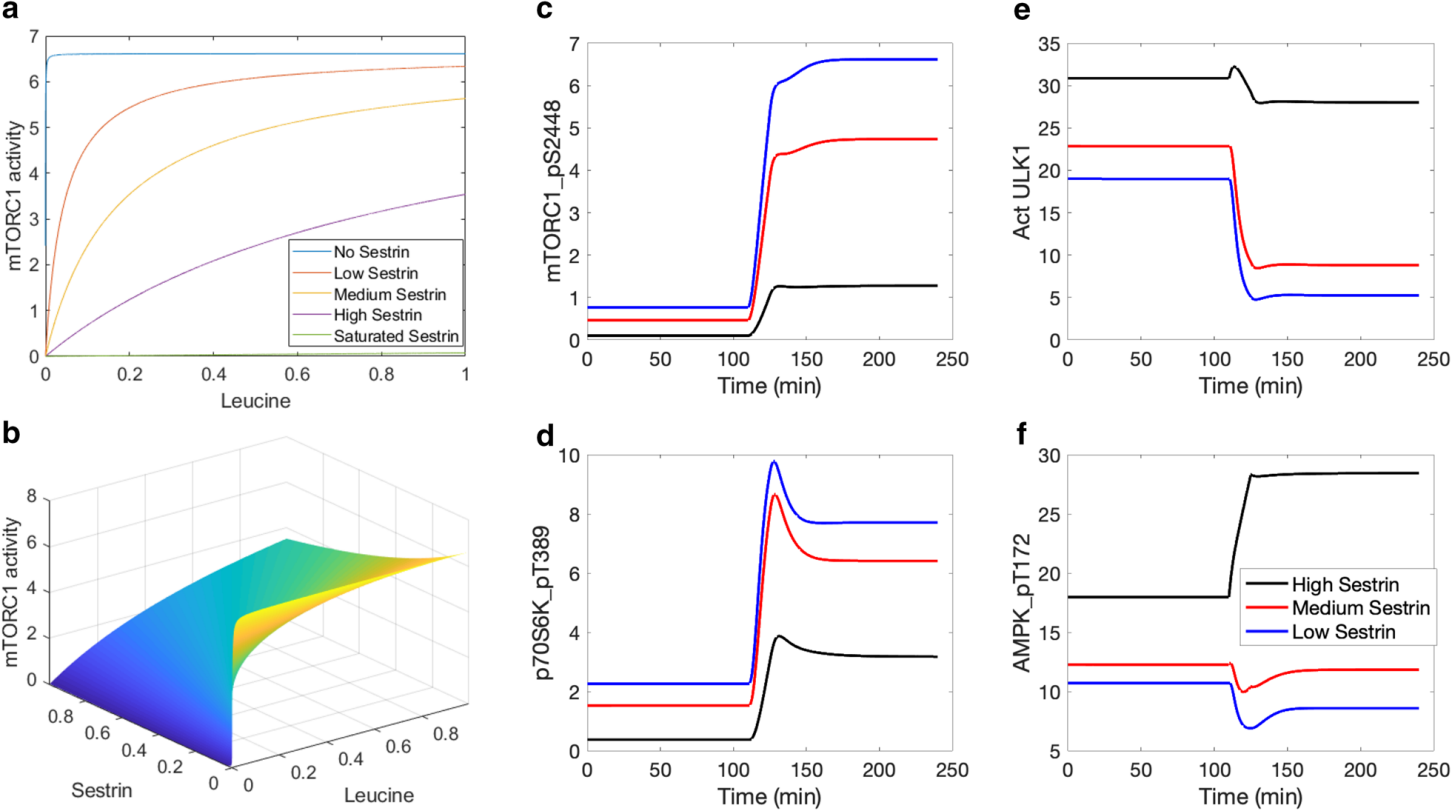
mTORC1 activity as a function of normalized leucine and sestrin2 concentrations, and model response to protein depletion and restoration. Panel **a**, mTORC1 activity for differing sestrin2 and leucine levels. Panel **b**, mTORC1 activity for the full range of leucine and sestrin2 concentrations. Predicted levels of mTORC1_pS2448 (**c**), p70S6K_pT389 (**d**), activated ULK1 (**e**), and AMPK_pT172 (**f**) under amino acid depletion (t < 120 min) and restoration (t > 120 min), obtained for differing sestrin2 levels

**Fig. 5:**
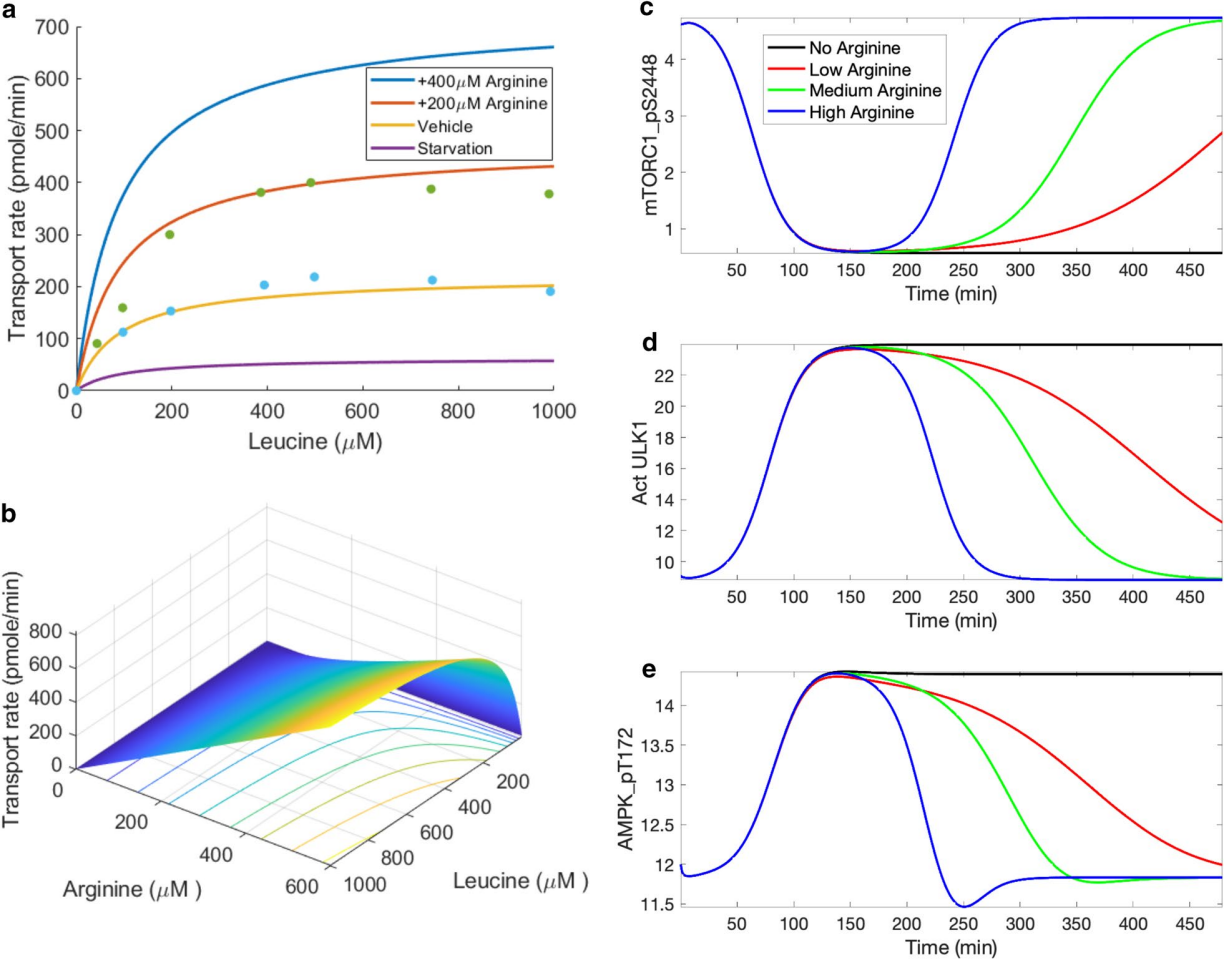
Leucine transport rate as a function of leucine and arginine concentrations, and its effect on mTORC1 reactivation. Panel **a**, leucine transport rate is computed with arginine concentration taken to be 50 µM (“Starvation”), 175 µM (“Vehicle”), 375 µM (“ + 200 µM Arginine”), 575 µM (“ + 400 µM Arginine”). Data taken from [17]. Panel **b**, leucine transport rate shown for the full range of leucine and arginine concentrations. Predicted levels of mTORC1 pS2448 (**c**), activated ULK1 (**d**), and AMPK_pT172 (**e**) under amino acid depletion, obtained for differing arginine levels

The regulation of mTORC1 activity by leucine and sestrin2 is shown in Fig. 4a,b, obtained by evaluating Eq. 4 at baseline AA and TSC1_TSC2 complex levels, over a range of leucine and sestrin2 concentrations based on [21]. Consider the system with a typical lysosomal leucine concentration. At a sufficiently low sestrin2 concentration, GATOR2 is activated, GATOR1 is inhibited, and mTORC1 activation rate is maximized. Conversely, at sufficiently high sestrin2 concentration, mTORC1 is rapidly dephosphorylated. Also, for a fixed sestrin2 concentration, increasing the concentration of leucine raises the phosphorylation rate of mTORC1. Now for a typical sestrin2 concentration, Fig. 5a exhibits the Michaelis–Menten-like dependence of mTORC1 activity on leucine.

Sestrin has been found to induce autophagy during diverse environmental stresses that provoke mitochondrial dysfunction [35], through AMPK activation and mTORC1 inhibition. Specifically, after leucine binds to sestrin2, the resulting complex activates AMPK. Thus, the model assumes that AMPK phosphorylation rate is proportional to the concentration product leucine and sestrin2 [36, 37]. *How does the interaction between sestrin2 and AAs affect the dynamics of mTORC1 inhibition AMPK activation, and autophagy stimulation?* To answer that question, we conduct simulations in which AA levels (including leucine) are initially set to 10% of baseline values, then subsequently increased to baseline levels. Simulations are conducted for high, medium, and low sestrin2 levels.

Key model variables are exhibited in Figs. 4 and Additional file 2: S5. Time profiles of mTORC1_S2448 and p70S6K_pT389 approximate that of AAs, whereas activated ULK1 exhibits the opposite trend. As sestrin2 concentration decreases, the activating effect of AAs on mTORC1 and p70S6K is enhanced (Fig. 4c,d), as is their inhibition of ULK1 and autophagy (Fig. 4e). Interestingly, AAs have competing effects on the phosphorylation of AMPK. Leucine and sestrin2 promote the phosphorylation of AMPK. In addition, through mTORC1 and its inhibition of IRS_p, AAs inhibit AMPK. That inhibition is modulated by sestrin2 (Fig. 4f). The model predicts that the latter (inhibition) dominates, resulting in the activation of AMPK when proteins are depleted; this result is consistent with experimental observations [38].

The next set of simulations, we focus on the modulation of mTORC1 by two major AA regulators, arginine and leucine. During AA starvation, lysosomal AAs (leucine in particular) migrate to the cytoplasm to facilitate mTORC1 activation. The transfer of lysosomal leucine to cytoplasm is mediated by SLC38A9, an arginine-regulated AA transporter.

[39]. We first determine how that transfer process varies as a function of lysosomal amino acide content. Wyant et al. reported that arginine enhances the capacity of SLC38A9 to transport leucine, by increasing its V_max_ without significantly affecting its K_m_ [17]. Taking both leucine and arginine into account, we model leucine transport rate, denoted V_max,L_, as

5 $$V_{{\text{max,L}}} = V_{{\text{max,L}}}^{*} \left( {\left[ A \right] + c_{A} } \right)\left( {\frac{\left[ L \right]}{{K_{M,L} + \left[ L \right]}}} \right)$$

where c_A_ denotes the contribution of lysine, which also transports leucine albeit significantly less effectively than arginine. In the absence of either AA groups ([A] + c_A_ = 0 or [L] = 0), there is no leucine efflux. Under basal conditions, the concentration of lysine is sufficiently low that c_A_ is taken to 0. The resulting leucine efflux, determined for a range of lysosomal leucine and arginine concentrations [21], is shown in Fig. 4(a,b). The predicted leucine transport rate at basal and + 200 μM arginine profiles are consistent with measurements reported by Wyant et al. [17] (Fig. 4a).

To assess the role of arginine in the regulation of mTORC1 and autophagy during AA depletion, we conducted simulations in which cytoplasmic AA progressively decreases during the first 3 h; afterwards, lysosomal AAs are released [40]. We simulate high, medium, low, and zero arginine levels by varying the rate at which cytoplasmic AA increases due to lysosomal leucine efflux. In the presence of insulin, AAs activate mTORC1, which inhibits autophagy by phosphorylating ULK1 [24]. Upon AA depletion, mTORC1 activation on the lysosomal surface is no longer maintained (Fig. 5c). Consequently, ULK1 Ser757 is rapidly dephosphorylated (Fig. 5d) [24], resulting in the activation of the ULK1 kinase and concomitant autophagy induction. A similar trend is observed for AMPK (Fig. 5e). When cytoplasmic AAs are sufficiently depleted, leucine is transported out of the lysosome by arginine-stimulated SLC38A9, activating mTORC1, phosphorylating p70S6K and ULK1, and suppressing autophagy (Fig. 5c,d). Additional results can be found in Additional file 2: Fig. S4. These results are consistent with reports by Yu et al. [40].

### Pharmacological activation of SIRT1

Resveratrol, a SIRT1 activator, mimics the anti-ageing effects of calorie restriction in lower organisms and mice, and leads to improved exercise performance and insulin sensitivity [41]. Other SIRT1-activating compounds (STACs) have been identified that are structurally unrelated to and more potent than resveratrol [41]. In the next set of simulations, we seek to unravel the metabolic molecular processes that give rise to both the health benefits and potential side effects of STACs.

**Fig. 6:**
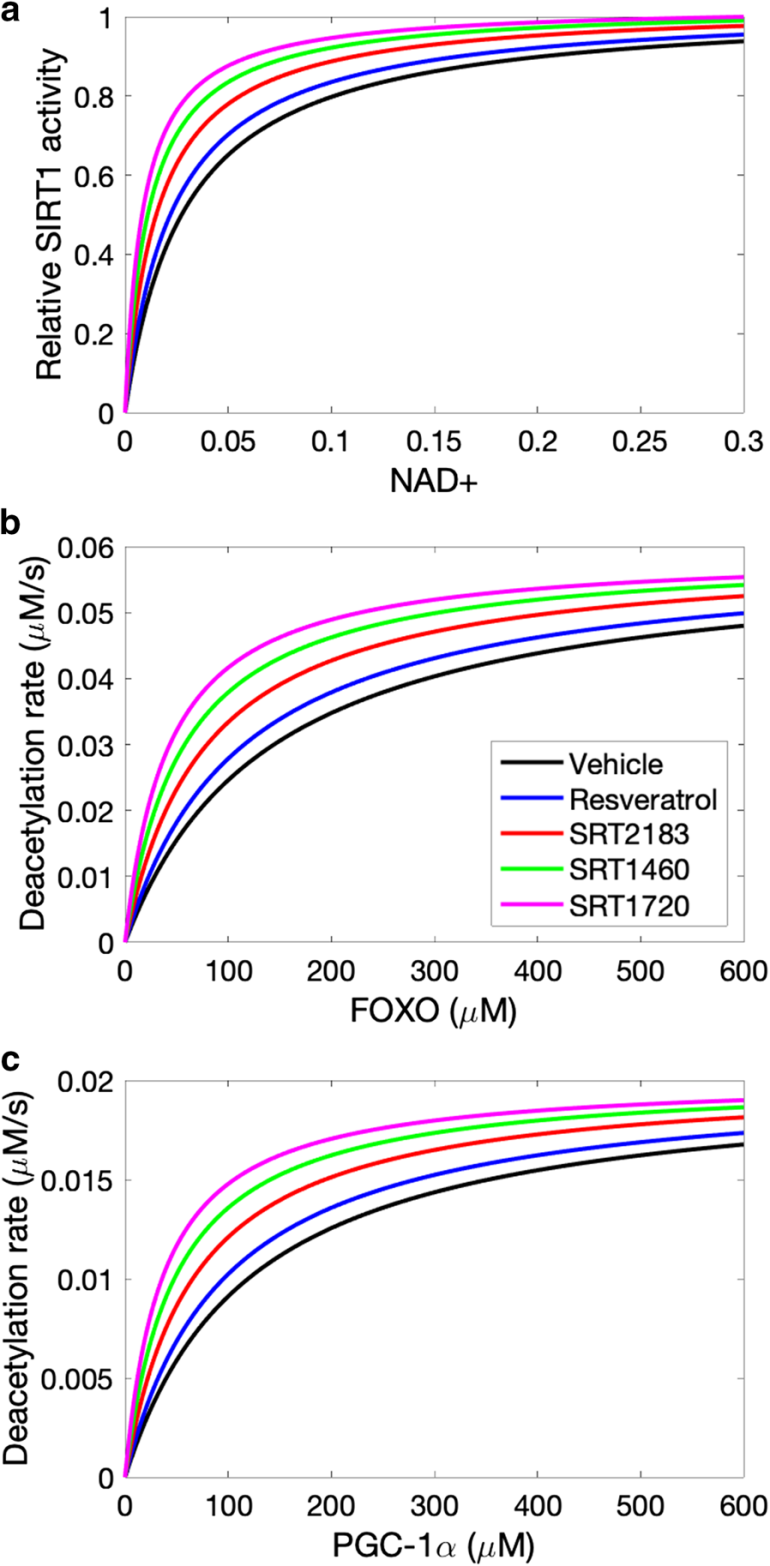
STACs activate SIRT1 (normalized values shown) by lowering the Michaelis–Menten constant for NAD+ (panel **a**). The deacetylation rates of FOXO and PGC-1α are subsequently affected (panels **b** and **c**)

*STACs and their anti-ageing effects*. We compare the effects of resveratrol to three other STACs: SRT2183, SRT1460, and SRT1720. We assume that the STACs alter the dependence of SIRT activity on NAD+ , which is described by Michaelis–Menten kinetics [20]. Based on in vitro findings by Milne et al. at 10 μM, resveratrol reduces baseline substrate K_m_ by 20%, SRT2183 by 50%, SRT1460 by 60%, and SRT1720 by 70% (Fig. 2a in Ref. [42]). As shown in Fig. 6a, at baseline K_m_ of [NAD+] = 0.029 mM, resveratrol slightly enhances SIRT1 activity by 11%. Other STACs has larger effects; SRT2183 elevates SIRT1 activity by 29%, SRT1460 by 43%, and SRT1720 by 54%.

As shown in Fig. 6a, the STACs increase SIRT1 activities to different degrees. SIRT1 transcription by deacetylating transcription factors FOXO [43]. The deacetylation rate of FOXO is described by Michaelis–Menten kinetics [19]. Model predicts that all STACs increase FOXO deacetylation but to significantly different degrees (Fig. 6b). At baseline K_m_ of [FOXO] = 141 µm [19], resveratrol increases FOXO deacetylation rate by a moderate degree (11%). Other STACs are more effective, with the largest increase obtained for SRT1720 (54%).

STACS activate AMPK and SIRT1, leading to the deacetylation of peroxisome proliferator-activated receptor-γ coactivator 1-α (PGC-1α). Similar to FOXO, the deacetylation rate of PGC-1α is also described by Michaelis–Menten kinetics [19]. PGC-1α activation improves mitochondrial biogenesis. At baseline PGC-1α K_m_ level, resveratrol increases PGC-1α deacetylation rate by 12%. Larger improvements are obtained for other STACs (Fig. 6c).

**Fig. 7:**
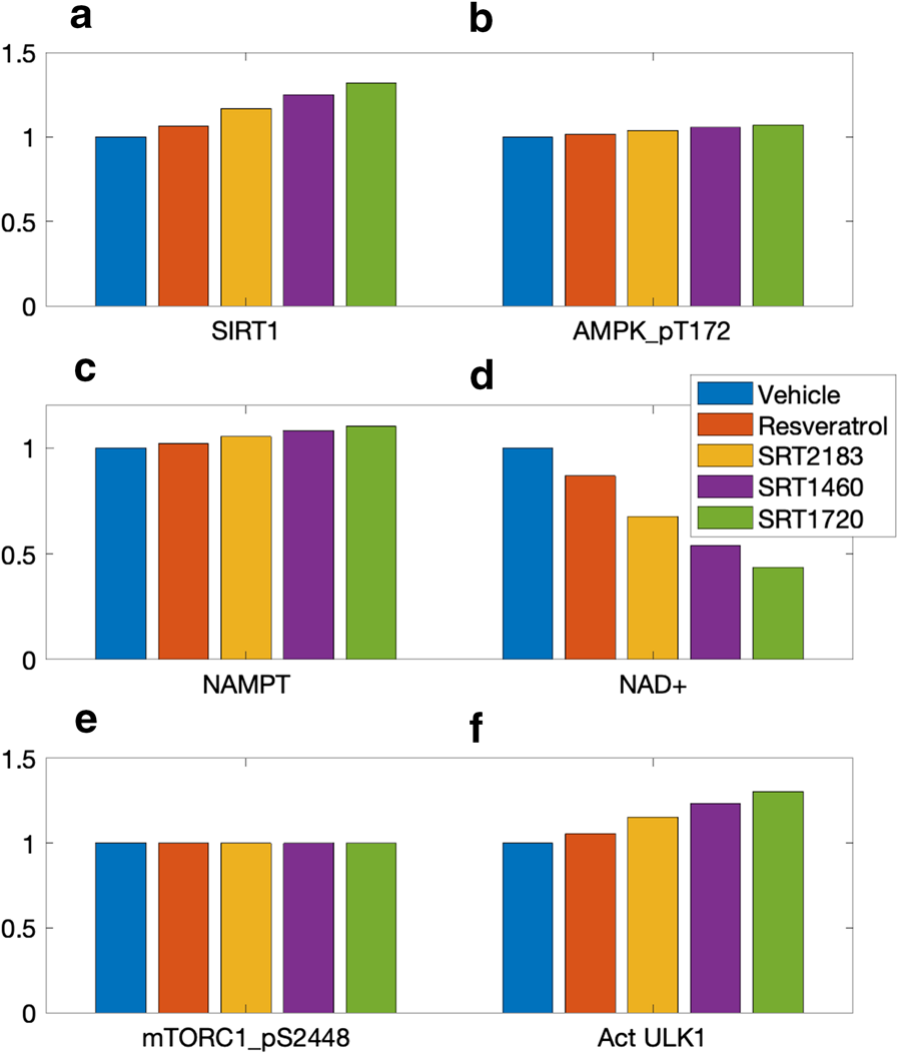
Predicted effects of STACs on SIRT1 activity (panel **a**), AMPK_pT172 (**b**), NAMPT (**c**), NAD+ (**d**), mTORC1_pS2448 (**e**), and activated ULK1 (**f**). Values are shown relative to control

The effects of these compounds on key protein activities are shown in Fig. 7. Consistent with results in Fig. 6a, STACs raise SIRT1 levels, by as much as 32% (SRT1720; Fig. 7a). SIRT1 in turn activates AMPK, although the SIRT1-induced increase in AMPK_pT172 is relatively small compared to SIRT1 (Fig. 7b). Phosphorylation of AMPK by resveratrol has been reported to be significant [44] or negligible [45], and may indeed be cell-type specific [46].

AMPK regulates nicotinamide phosphoribosyl transferase (NAMPT) [47], which acts as a limiting enzyme in the conversion of NAM to nicotinamide mononucleotide (NMN). Taken in isolation, the STAC-induced elevation in NAMPT (Fig. 7c) would accelerate the conversion of nicotinamide (NAM) to NMN, resulting in an increase in the downstream NAD+ . However, the STACs also accelerate the consumption of NAD+ by binding on the allosteric site of SIRT1. Indeed, the enhanced SIRT1 consumption is the stronger effect, resulting in lower NAD+ following the administration of STACs (Fig. 7d), by as much as 56% with SRT1720.

The predicted minimal increase in AMPK by STACs results in a negligible effect on mTORC1 inhibition (Fig. 7e). Nonetheless, the beneficial effects of STACs may manifest through AMPK’s direct phosphorylation of ULK1 and the regulation of autophagy [24]. Despite mTORC1’s insensitivity to STACs, the level of activated ULK1 is predicted to be increased by STACs (Fig. 7f).

**Fig. 8:**
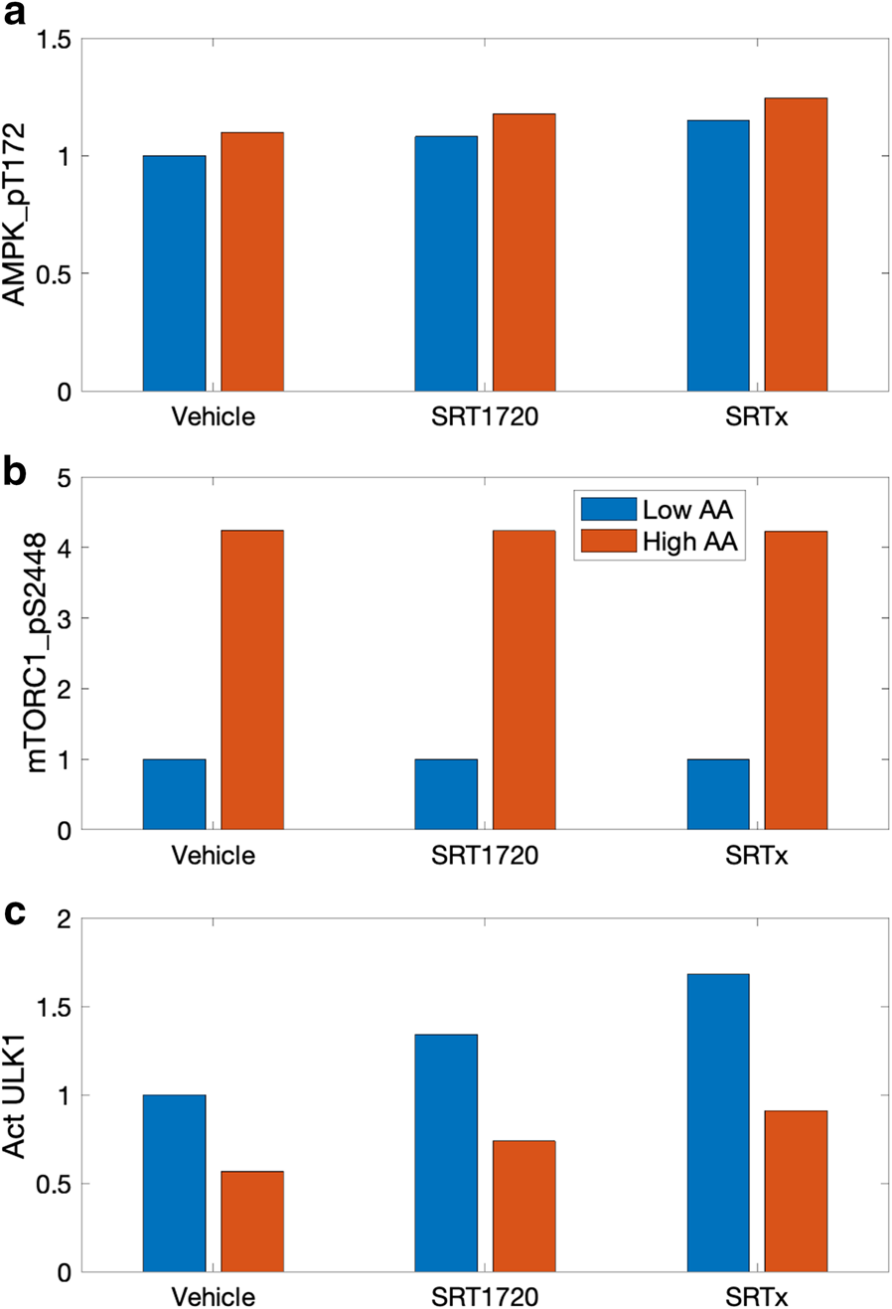
Potential overactivation of autophagy by STACs at high nutrient level. **a** normalized levels of AMPK_pT172 obtained for control (“Vehicle”), for SRT1720, where SIRT1 substrate K_m_ is reduced by 70%, and for a hypothetical STAC where K_m_ is reduced by 90% (“SRTx”). For each case, AMPK_pT172 levels are determined at an AA level is 25% of control (“Low AA”) and for reference AA conditions (“High AA”). **b** normalized levels of mTORC1_pS2448. ***c***, activated ULK1. With a sufficiently strong STAC (“SRTx” and “High AA”), autophagy may be activated at a level close to that at AA deprivation (compare with “Vehicle” and “Low AA”)

Given that STACs enhance AMPK and ULK1, we investigate the following possibility: *Can STAC intake lead to premature autophagy, i.e., protein catabolism under an abundance of nutrients?* The inhibitory phosphorylation of ULK1 by mTORC1 serves as a negative feedback to prevent overactive autophagy and to avoid a futile cycle in which newly synthesized cellular building blocks are prematurely broken down again. But is this safeguard sufficient under the actions of STACs? To answer these questions, we consider three groups: control, SRT1720, and a (hypothetical) higher-performing STAC (called “SRTx”) which we assume reduces substrate K_m_ by 90%. Simulations are conducted for baseline AA level, and a low AA level that is 25% of baseline. Predicted AMPK_pT172, mTORC1_pS2448, and ULK1 activation levels are shown in Fig. 8. STAC and AA both increase AMPK_pT172, whereas mTORC1_pS288 is substantially elevated by AA, but is relatively insensitive to STAC. ULK1 is activated by AMPK (Fig. 1), and thus simulations predict that STAC increases activated ULK1 level. In contrast, ULK1 is inhibited by mTORC1, and thus AA abundance is predicted to suppress ULK1. Simulation results indicate that, with abundance of nutrients, SRTx yields ULK1 activation level that is 91% of the analogous level obtained for the control group under AA deprivation. The similar autophagy activation under these two sets of conditions, despite the widely differing AA abundance levels, suggests that a sufficiently potent STAC may induce premature autophagy [1].

### Parameter sensitivity analysis

**Fig. 9:**
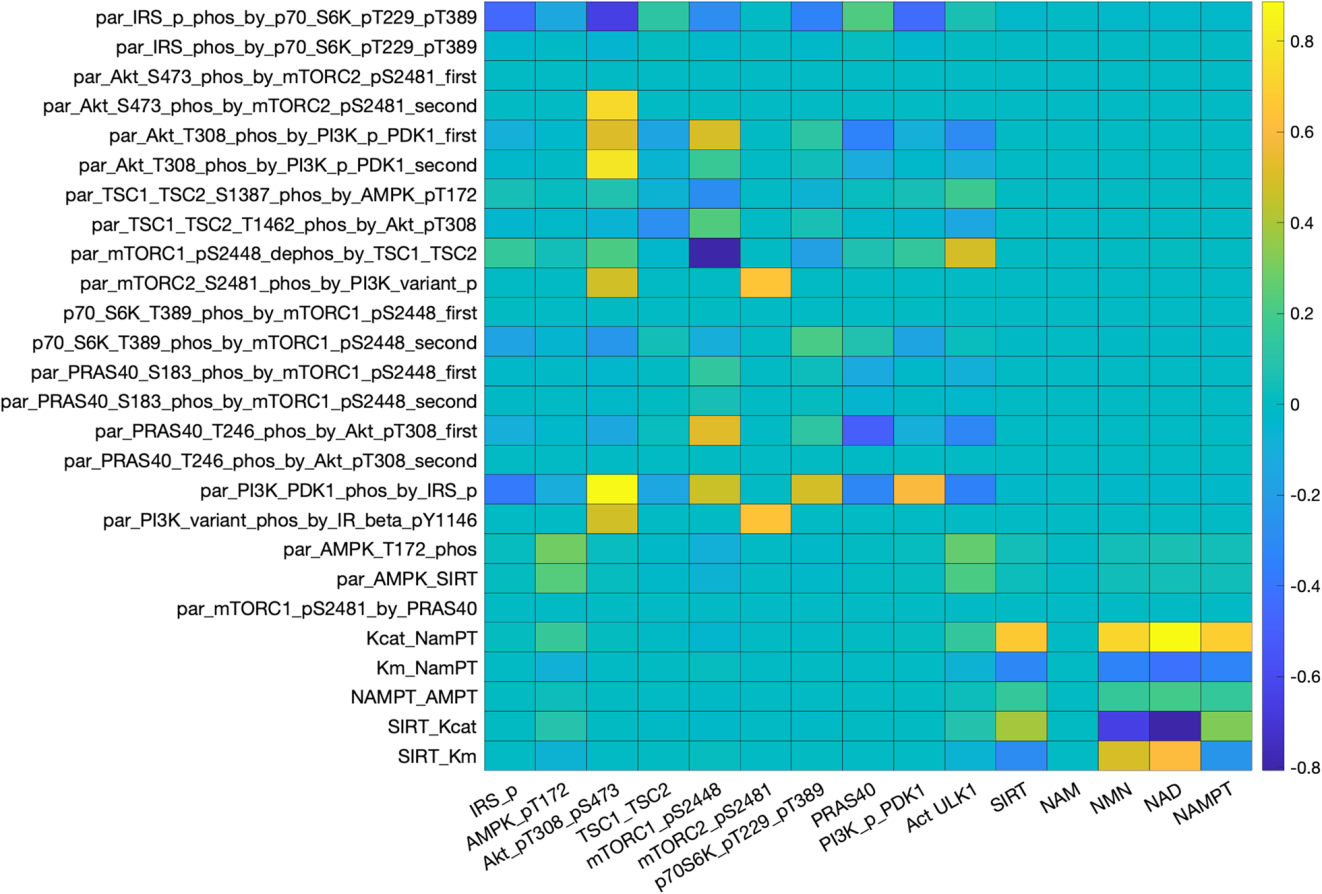
Heat map that illustrates the sensitivity of key model outputs (horizontal axis) to local variations in selected model parameters (vertical axis). Definition of the parameters can be found in the Supplemental Materials

We perform local sensitivity analysis to assess the response of model outputs to small variations in selected parameter values (Fig. 9). Two distinguishable heat-map regions are identified with mostly non-zero entries. These regions are associated with the original model components: the insulin/IGF-1 signaling pathway (top-left region) and the Preiss-Handler and salvage signaling pathways (bottom-right region). These two components are coupled via the effects of AMPK on NAMPT, and of SIRT on AMPK. Variations in parameters in one region have typically minor (but nonzero) effects on outputs in the other region.

Within the insulin/IGF-1 signaling pathway, model outputs are particularly sensitive to variations in links that activate or inhibit proteins that have a large number of downstream effects, e.g., the activation of IRS_p by p70S6K_pT229_pT389 (“par_IRS_phos_by_p70_S6K_pT229_pT389”). Except for mTORC2_pS2481, other outputs in the insulin/IGF-1 signaling pathway exhibit significant sensitivity to variations in that activation strength, because IRS_p directly modulates major model variables such as PI3K_PDK1 and AMPK. In contrast, model solution is insensitive to changes in the activation of IRS_pS636 by p70S6K_pT229_pT389, in large part because the IRS_pS636 has no direct downstream effect.

Within the Preiss-Handler and salvage signaling pathways, most model outputs exhibit significant sensitivity to variations in the selected parameters associated with the pathways. NAM exhibits remarkable stability, because of the robust negative feedback cycle involves itself, NMN, NAD+ , and SIRT1. Proteins in this pathway are particularly sensitive to changes in the abundance of active NAMPT (“Kcat_NAMPT”). This result suggests that an NAMPT activator may be a promising pharmacological approach for raising intracellular NAD+ and SIRT1, to realize diverse and potentially impactful therapeutic benefits.

## Discussion

The present study describes a state-of-the-art computational model for investigating essential signaling pathways in growth, ageing, metabolism, and disease in mammals. Major model components include the insulin/IGF-1 or mTOR signaling pathway [15], the Preiss-Handler and salvage pathways [16], energy sensor AMPK, and transcription factors FOXO and PGC-1α. The mTOR signaling pathway couples energy and nutrient abundance to the execution of cell growth and division. That function can be attributed to the ability of mTORC1 to sense energy, nutrients, and growth factors, by regulating other important kinases, such as S6K and AKT. The Preiss-Handler and salvage pathways regulate the metabolism of NAD+ as well as NAD+ -consuming proteins such as sirtuins. Key findings include:

- Model simulations indicate that simultaneously inhibiting AKT or PI3K_PDK1, and inhibiting mTORC1 effectively suppresses PRAS40 phosphorylation on both Ser183 and Thr246 sites, further enhancing the inhibition of mTORC1 by PRAS40 (Fig. 1). This result suggests a clinically important role of PRAS40 in controlling tumor growth.
- We provide the first computational model that capitulates the interplay between sestrin2 and leucine, and the effect on mTORC1 activity. With a crucial role in metabolic regulation through the activation of AMPK and inhibition of mTORC1, sestrin2 might serve as a therapeutic target for cancers, metabolic diseases, and neurodegenerative diseases.
- Given that sestrin2’s inhibitory effect on mTORC1 activity can be significantly impacted by AAs such as leucine (Fig. 5), dietary modifications may enhance the efficacy of therapies that target mTORC1 and sestrin2.
- The model captures the interactions between arginine and leucine during protein deprivation, and predicts a signal that reactivates mTORC1 and downregulates autophagy (Fig. 4).
- The model capitulates the regulation of autophagy by SIRT1. Simulations indicate that, by activating SIRT1, STACs in sufficiently high dosages may lead to premature autophagy (Fig. 8).

mTORC1 signaling is switched on by a number of oncogenic signaling pathways and may be hyperactive in up to 70% of all human tumors [48]. Moreover, mTORC1 is known to increase lifespan in diverse organisms ranging from yeast to mammals [1]. Thus, there is much interest in targeting mTORC1 signaling as a potential therapeutic avenue for anti-cancer therapy. Rapamycin, originally developed as an immunosuppressant that targets T-cells, is arguably the best known mTORC1 inhibitor and has been shown to extend lifespan in mice [49]. However, despite its specificity, rapamycin in typical dosages does not completely inhibit all mTORC1 activities [50], limiting its efficacy as an anti-cancer agent. In fact, cancer patients whose tumors exhibit a mutational activation of PI3K/AKT signaling have a low response rate for rapamycin and its rapalogs (e.g., breast, colon and prostate cancer, and glioblastoma [51, 52]). This inadequate therapeutic response is believed to result from rapamycin and its rapalogs’ incomplete inhibition of mTORC1-mediated phosphorylation of 4E-BP1 and a concomitant activation of AKT via loss of a negative feedback mechanism [15].

Mi et al. suggested that the acquired resistance to rapamycin in cancer cells may be attributable to the redundant phosphorylation of PRAS40 by both AKT and mTORC1 signaling [53]. Also, while PI3K inhibitors such as wortmannin are potential anti-cancer agents on their own [54], a more nearly complete inhibition of mTORC1 may be achieved when inhibitors of the PI3K/AKT pathway are administered in conjunction with rapamycin (Fig. 1). These results suggest a potentially important role of PRAS40 in the translational control of tumor progression. Indeed, as illustrated in our model simulations (Fig. 2), dual inhibition of PI3K and mTORC1 signaling by rapalogs in combination with PI3K or AKT inhibitors has demonstrated profound efficacy in preclinical cancer models [55–58].

Chronic administration of rapamycin leads to insulin resistance due to its suppression of mTORC2 [26]. Sebastian and co-workers demonstrated intermittent administration of rapamycin (e.g., once every 5 days) mitigates its detrimental effect on glucose homeostasis [33, 34]. Our model simulations indicate that an optimal rapamycin dosage can be identified which, in chronic and continuous usage, attenuates the detrimental effect on insulin sensitivity while preserving rapanycin’s anti-cancer and anti-ageing effects via mTORC1 inhibition (Fig. 3).

In some lung, breast, ovarian cancers and in glioblastomas [59], mTORC1 is hyperactived and these cancer cells have been shown to be hypersensitive to rapamycin. Thus, the associated cancers may be particularly amenable to therapeutic strategies that limit mTORC1 activity. Sestrin2 negatively regulates mTORC1 and appears to function as a tumor suppressor [60, 61], and may also serve as a potential therapeutic target for ageing, metabolic and neurodegenerative diseases [62]. The present model provides the first computational platform that capitulates the crucial regulation of mTORC1 by sestrin2, and its modulation of leucine. During acute starvation, sestrin2 binds to GATOR2 and impedes the latter’s inhibition of GATOR1, resulting in the suppression of mTORC1. By binding with sestrin2, leucine promotes the downstream inhibition of GATOR1 and enhances mTORC1 activation. These interactions are summarized in Fig. 5. Given that sestrin2’s inhibitory effect on mTORC1 activity can be significantly impacted by AAs such as leucine, dietary modifications may enhance the efficacy of anti-cancer therapies and other disease treatments that target mTORC1 and sestrin2.

Another arginine sensor is the lysosomal AA transporter SLC38A9. Arginine stimulates SLC38A9 and promotes its interaction with the Rag GTPase-Regulator complex, which results in the activation of mTORC1. Wyant et al. reported that a mutant of SLC38A9 that does not interact with arginine lacks the ability to signal AA sufficiency to mTORC1 [17]. Another important role of SLC38A9 in AA homeostasis is to transport AAs produced by lysosomal proteolysis, such as leucine, from lysosomes to the cytosol, thereby reactivating mTORC1. The critical role of SLC38A9 in this process is evinced by the SLC38A9-null HEK293T cells, which exhibit whole-cell AA levels similar to wild-type, but significantly higher lysosomal AA concentrations including leucine [17]. The efflux of lysosomal leucine and the subsequent activation of mTORC1 may regulate autophagy. During starvation, mTORC1 is inhibited, which attenuates its inhibitory phosphorylation of ULK1 and promotes autophagy. Thus, intracellular nutrients produced by autophagy can stimulate mTORC1 signaling and provide a negative feedback signal to downregulate autophagy (more below) [40]. The present model is unique in that it explicitly and separately represents arginine and leucine, rather than lumping them into a single AA category. Consequently, the model captures the exquisite interactions of these AAs, and the resulting signal to the mTORC1 pathway and its reactivation during protein deprivation. Indeed, the model is the first to investigate the reactivation of mTORC1 by proteolysis following autophagy (Fig. 4) [40].

STACs enhance autophagy via its activation of SIRT1, followed by the activation of AMPK and inhibition of the mTORC1. The elevated SIRT1 activity also facilitates the deacetylation of FOXOs, which are transcription factors that have major impacts on longevity and cancer. In the absence of growth factors or AAs, the reduction in activated AKT leads to the dephosphorylation of FOXOs and drives their relocalization from the cytoplasm to the nucleus. The subsequent deacetylation of FOXOs by SIRT1 mediates stress resistance response. AMPK phosphorylation also increases the transcription activity of FOXOs. Emerging evidence suggests that FOXO factors act as a tumor suppressor in a variety of cancers [63]. The model represents mTORC1 inhibition by AMPK via its stimulation of ULK1 and TSC1_TSC2. AMPK is also known to suppress mTORC1 by phosphorylating Raptor [64]. That direct inhibition of mTORC1 is not represented explicitly in the present model, but may be important in conditions involving TSC1_TSC2 mutation.

Additionally, SIRT1 regulates autophagy through the direct deacetylation of autophagy-related genes such as ATG. Studies have demonstrated an essential role for SIRT1 in the induction of autophagy [65, 66], as well as the protective effects of the SIRT1-induced autophagy in preventing or attenuating neurotoxicity [67, 68]. However, a notable model result is that by activating SIRT1, STACs may lead to the decoupling of the autophagic response from the organisms’ nutrient and energy status. Model simulations suggest that a sufficiently potent STAC may yield premature autophagy (Fig. 8), in which newly synthesized cellular building blocks are prematurely and unnecessarily catabolized despite an abundance of nutrients.

The insulin/IGF-1 signaling pathway of the present model is fitted primarily for C2C12 cells [11], whereas the Preiss-Holder and salvage pathway is formulated primarily using non-specific mammalian cell data [13]. Thus, a limitation of the model is that, while many model metabolic pathways are ubiquitous, some regulatory kinetics and interactions may vary among cells. For instance, SIRT1 is activated by starvation in most cells [69], but in the mouse pancreas, SIRT1 is inactivated by the reduction in the NAD+/NADH ratio [70]. AMPK appears to have only a limited regulatory effect on mTORC1 in C2C12 cells, but is known to directly inhibit mTORC1 in HEK293T cells [71]. Another limitation is that while the model reproduces cell culture experimental results, its predictions may well deviate from in vivo observations.

Its limitations notwithstanding, the present model can be extended and applied to investigations of growth, ageing, metabolism, and disease in mammals. A worthwhile future extension is to study how the key regulatory pathways differ between the sexes: In the mouse, sex differences have been reported mTORC1 and mTORC2 activity levels [72]. Another future direction is the age-dependent interactions among the regulatory pathways. In human skeletal muscle, mTORC1 is activated under different conditions depending on the age of the individuals [73, 74]. Similarly, marked changes have been reported in SIRT1 activity and NAD+ levels as individuals age [75, 76]. The development of models that take into account cell or tissue specificity, together with sex and age, can help identify therapeutic strategies that target metabolic pathways.

## Conclusions

Traditionally, the investigation of human ageing and disease has relied on cell cultures and animal models, including non-vertebrates (e.g., yeast, worm, and fly) and vertebrates (e.g., zebrafish, mice, dogs, and primates), as well as clinical trials. With the advent of bioinformatics and computational biology, computational modeling and analysis techniques can provide accurate simulation of biological processes. In sum, we have developed a state-of-the-art in silico model for investigating the interactions among signaling pathways and environmental stimuli in growth, ageing, metabolism, and diseases. Major findings include the clinically important role of PRAS40 and diet in mTORC1 inhibition, and a potential link between SIRT1-activating compounds and premature autophagy. Additionally, the model captures the synergy among leucine, sestrin2, and arginine, and how their interactions regulate the mTORC1 pathway. The model can be used as an essential component to simulate (1) gene manipulation, (2) therapies for cancer, metabolic diseases, and neurodegenerative diseases, (3) calorie restrictions, and (4) chronic stress. Simulation results can be interpreted to assess the implications on longevity and ageing‐related diseases.

## Supplementary Information

**Additional file 1**. TableS1. Model equations.

**Additional file 2.** Figure S1. Schematic diagram depicting connections among model components that form the signaling pathways.  Figures S2 and S3. Effects of insulin, rapamycin, and wortmannin on key proteins and their interactions. Figure S4. Effect of protein deprivation and subsequent leucine efflux on key model variables, obtained for differing arginine levels. Figure S5. Effect of protein depletion and restoration on key model variables, obtained for differing sestrin2 levels.

## Data Availability

MATLAB programs and model parameters that are used in the model simulations all can be accessed at https://github.com/MehrshadSD/A-Computational-Model-for-Cell-Energy-Balance-and-Metabolism

## Abbreviations

AA: Amino Acid
AMPK: Adenosine Monophosphate-activated Protein Kinase
GATOR: Gap Activity TOward Rags
FOXO: FOrkhead boX O
IGF-1: Insulin-like Growth Factor 1
IRS: Insulin Receptor Substrate
LKB1: Liver Kinase B1
mTOR: Mechanistic Target Of Rapamycin
NA: Nicotinic Acid
NAD+: Nicotinamide Adenine Dinucleotide
NAM: NicotinAMide
NAMPT: NicotinAMide PhosphoribosylTransferase
NMN: Nicotinamide MonoNucleotide
NMNAT: Nicotinamide MonoNucleotide AdenylylTransferase
PDK1: Pyruvate Dehydrogenase Kinase 1
PGC-1α: Peroxisome proliferator-activated receptor Gamma Coactivator 1-alpha
PI3K: PhosphoInositide 3-Kinases
PRAS: Proline-Rich AKT substrate of 40 kDa
SIRT: Silent Information RegulaTor
STAC: SIRT1-Activating Compound
TSC: Tuberous Sclerosis Complex
ULK1: Unc-51 Like autophagy activating Kinase 1
