# Supplementary material for: Interactions among mTORC, AMPK and SIRT: a computational model for cell energy balance and metabolism
Source: Cell Commun Signal. 2021 May 20;19:57. doi: 10.1186/s12964-021-00706-1 (PMC8135154; doi:10.1186/s12964-021-00706-1)
Supplement: Supplementary file 3 — Additional file 2. Figure S1. Schematic diagram depicting connections among model components that form the signaling pathways. Figures S2 and S3. Effects of insulin, rapamycin, and wortmannin on key proteins and their interactions. Figure S4. Effect of protein deprivation and subsequent leucine efflux on key model variables, obtained for differing arginine levels. Figure S5. Effect of protein depletion and restoration on key model variables, obtained for differing sestrin2 levels. [file 12964_2021_706_MOESM3_ESM.docx]

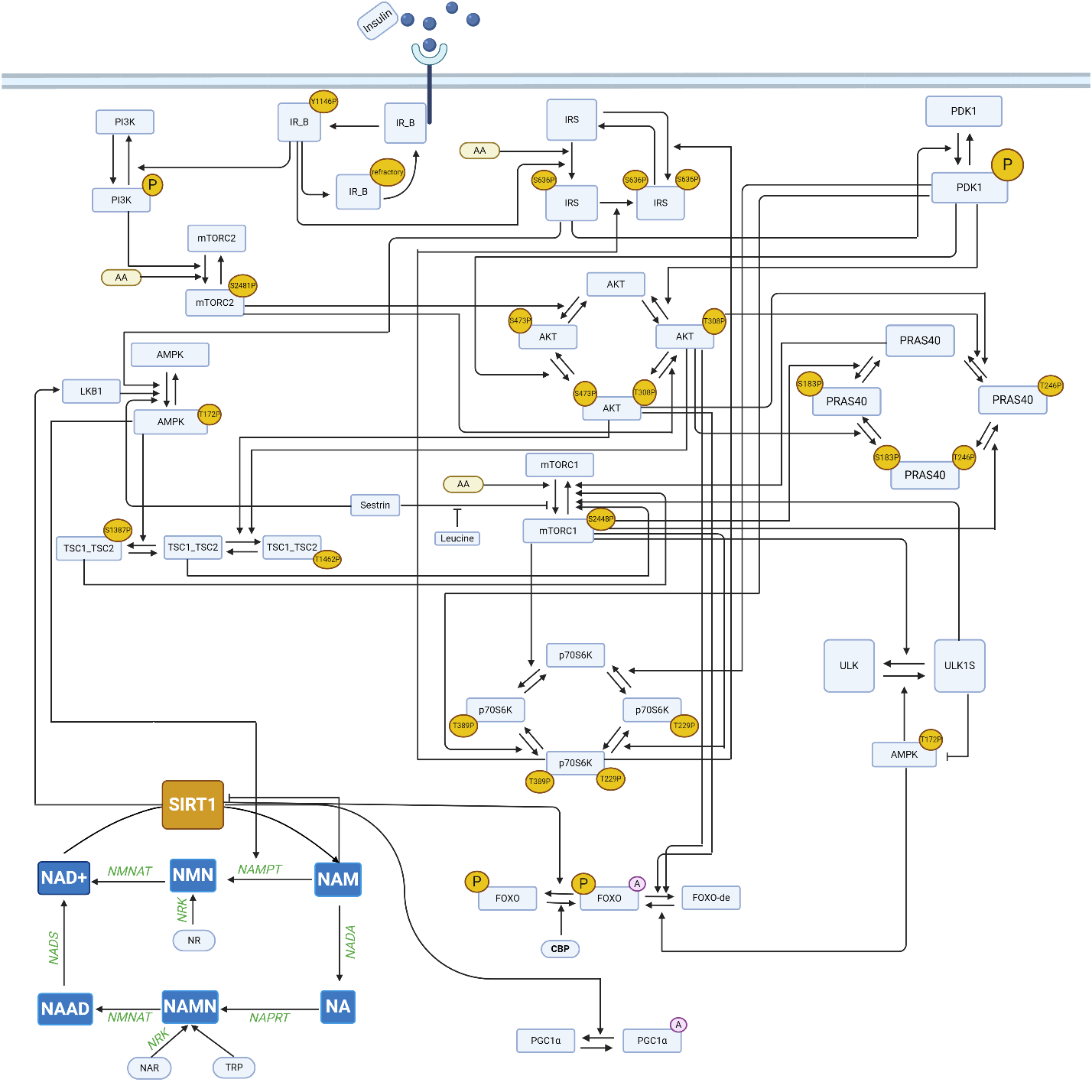


**Figure S1.** Schematic diagram depicting connections among model components that form the signaling pathways. AA represents amino acids; “A” in purple circles denote the acetylated form of the complex; “P” in yellow circles denote the phosphorylated form.

**Table S1**. Model equations.

| $\frac{\boldsymbol{d}\left[ \boldsymbol{IR\beta} \right]}{\boldsymbol{dt}}$  $\frac{\boldsymbol{d}\left[ \boldsymbol{IR\beta\_pY}\boldsymbol{1146} \right]}{\boldsymbol{dt}}$  $\frac{\boldsymbol{d}\left[ \boldsymbol{IR\beta\_refractory} \right]}{\boldsymbol{dt}}$  $\frac{\boldsymbol{d}\left[ \boldsymbol{mTORC}\boldsymbol{1\_ pS}\boldsymbol{2448} \right]}{\boldsymbol{dt}}$  $\frac{\boldsymbol{d}\left[ \boldsymbol{mTORC}\boldsymbol{1} \right]}{\boldsymbol{dt}}$  $\frac{\boldsymbol{d}\left[ \boldsymbol{mTORC}\boldsymbol{2} \right]}{\boldsymbol{dt}}$  $\frac{\boldsymbol{d}\left[ \boldsymbol{mTORC}\boldsymbol{2\_pS}\boldsymbol{2481} \right]}{\boldsymbol{dt}}$  $\frac{\mathbf{d}\left[ \mathbf{IRS} \right]}{\mathbf{dt}}$  $\frac{\mathbf{d}\left[ \boldsymbol{IRS\_p} \right]}{\mathbf{dt}}$  $\frac{\mathbf{d}\left[ \boldsymbol{IRS\_pS636} \right]}{\mathbf{dt}}$  $\frac{\mathbf{d}\left[ \boldsymbol{TSC1\_TSC2} \right]}{\mathbf{dt}}$  $\frac{\boldsymbol{d}\left[ \left( \boldsymbol{TSC}\boldsymbol{1\_TSC}\boldsymbol{2\_pT}\boldsymbol{1462} \right) \right]}{\boldsymbol{dt}}$  $\frac{\boldsymbol{d}\left[ \left( \boldsymbol{TSC}\boldsymbol{1\_TSC}\boldsymbol{2\_pS}\boldsymbol{1387} \right) \right]}{\boldsymbol{dt}}$  $\frac{\mathbf{d}\left[ \mathbf{PRAS40} \right]}{\mathbf{dt}}$  $\frac{\boldsymbol{d}\left[ \boldsymbol{PRAS}\boldsymbol{40\_pS}\boldsymbol{183} \right]}{\boldsymbol{dt}}$  $\frac{\boldsymbol{d}\left[ \boldsymbol{PRAS}\boldsymbol{40\_pT}\boldsymbol{246} \right]}{\boldsymbol{dt}}$  $\frac{\boldsymbol{d}\left[ \boldsymbol{PRAS}\boldsymbol{40\_pT}\boldsymbol{246\_pS}\boldsymbol{183} \right]}{\boldsymbol{dt}}$  $\frac{\mathbf{d}\left[ \mathbf{AKT} \right]}{\mathbf{dt}}$  $\frac{\mathbf{d}\left[ \boldsymbol{Akt\_pT308\_pS473} \right]}{\mathbf{dt}}$  $\frac{\mathbf{d}\left[ \boldsymbol{Akt\_pT308} \right]}{\mathbf{dt}}$  $\frac{\mathbf{d}\left[ \boldsymbol{Akt\_pS473} \right]}{\mathbf{dt}}$  $\frac{\mathbf{d}\left[ \mathbf{p70S6K} \right]}{\mathbf{dt}}$  $\frac{\boldsymbol{d}\left[ \boldsymbol{p}\boldsymbol{70}\boldsymbol{S}\boldsymbol{6}\boldsymbol{K\_pT}\boldsymbol{229} \right]}{\boldsymbol{dt}}$  $\frac{\boldsymbol{d}\left[ \boldsymbol{p}\boldsymbol{70}\boldsymbol{S}\boldsymbol{6}\boldsymbol{K\_pT}\boldsymbol{389} \right]}{\boldsymbol{dt}}$  $\frac{\boldsymbol{d}\left[ \boldsymbol{p}\boldsymbol{70}\boldsymbol{S}\boldsymbol{6}\boldsymbol{K\_pT}\boldsymbol{389\_pT}\boldsymbol{229} \right]}{\boldsymbol{dt}}$  $\frac{\boldsymbol{d}\left[ \boldsymbol{PI}\boldsymbol{3}\boldsymbol{K} \right]}{\boldsymbol{dt}}$  $\frac{\boldsymbol{d}\left[ \boldsymbol{PI}\boldsymbol{3}\boldsymbol{K\_p} \right]}{\boldsymbol{dt}}$  $\frac{\boldsymbol{d}\left[ \boldsymbol{PDK}\boldsymbol{1} \right]}{\boldsymbol{dt}}$  $\frac{\boldsymbol{d}\left[ \boldsymbol{PDK}\boldsymbol{1\_p} \right]}{\boldsymbol{dt}}$  $\frac{\boldsymbol{d}\left[ \boldsymbol{AMPK} \right]}{\boldsymbol{dt}}$  $\frac{\mathbf{d}\left[ \boldsymbol{AMPK\_pT172} \right]}{\mathbf{dt}}$  $\frac{\mathbf{d}\left[ \boldsymbol{FOXO\_de} \right]}{\mathbf{dt}}$  $\frac{\mathbf{d}\left[ \boldsymbol{FOXO\_AC\_P} \right]}{\mathbf{dt}}$  $\frac{\mathbf{d}\left[ \boldsymbol{FOXO\_P} \right]}{\mathbf{dt}}$  $\frac{\boldsymbol{d[PGC1\alpha]}}{\mathbf{dt}}$  $\frac{\boldsymbol{d[AC\_PGC1\alpha]}}{\mathbf{dt}}$  $\frac{\mathbf{d[ULK1]}}{\mathbf{dt}}$  $\frac{\boldsymbol{d[AC\_ULK1]}}{\mathbf{dt}}$  $\frac{\boldsymbol{d[Nam\_ex]}}{\mathbf{dt}}$  $\frac{\mathbf{d[NADbound]}}{\mathbf{dt}}$  $\frac{\mathbf{d[NR]}}{\mathbf{dt}}$  $\frac{\mathbf{d[NaAD]}}{\mathbf{dt}}$  $\frac{\mathbf{d[NMN]}}{\mathbf{dt}}$  $\frac{\mathbf{d[NA]}}{\mathbf{dt}}$  $\frac{\mathbf{d[NaMN]}}{\mathbf{dt}}$  $\frac{\mathbf{d[NAM]}}{\mathbf{dt}}$  $\frac{\mathbf{d[NAD]}}{\mathbf{dt}}$  $\frac{\mathbf{d[NAR]}}{\mathbf{dt}}$  **NMN_efflux**  **NAPRT**  **NMNAT1_NMN**  **NMNAT1_NaMN**  **NADS**  **NT5_NMN**  **PNP_NR**  **NNMT**  **Nam_efflux**  **NT5_NaMN**  **SIRT**  **NAD_consumption_without_Nam_inhibition**  **NADA**  **NAMimport**  **NAMPT**  **NAR_efflux**  **NA_efflux**  **NAD_binding**  **NRK1_NaMN**  **PNP_NAR**  **NR_efflux**  **NRK1_NMN** | $+\left( par\_IR\_beta\_ready*\left[ IR\beta\_refractory \right] \right)$  $-([IR\beta]*par\_IR\_beta\_phos\_by\_Insulin*[Insulin])$  $([IR\beta]*par\_IR\_beta\_phos\_by\_Insulin*[Insulin])$  $-(par\_IR\_beta\_pY1146\_dephos*[IR\beta\_pY1146])$  $+(par\_IR\_beta\_pY1146\_dephos*[IR\beta\_pY1146])$  $-(par\_IR\_beta\_ready*[IR\beta\_refractory])$  $-([TSC1\_TSC2]+[TSC1\_TSC2\_pS1387])*[mTORC1\_pS2448]*par\_mTORC1\_pS2448\_dephos\_by\_TSC1\_TSC2)$  $-(b\_pras\_mtorc1*0.00068*[mTORC1\_pS2448]*10^(0.25*[PRAS40]))$  $-([mTORC1\_pS2448]*[Act\_ULK1]*0.00016)$  $+ [Amino\_Acid]*[mTORC1]*par\_mTORC1\_S2448\_activation\_by\_Amino\_Acids$  $*( \frac{mTORC1_{vmax}*([leucine_{a}]+[leucine_{conc}])}{[leucine_{a}]+[leucine_{conc}]+[sestrin2]} )$  $+([TSC1\_TSC2]+[TSC1\_TSC2\_pS1387])*[mTORC1\_pS2448]*par\_mTORC1\_pS2448\_dephos\_by\_TSC1\_TSC2)$  $+(b\_pras\_mtorc1*0.00068*[mTORC1\_pS2448]*10^(0.25*[PRAS40]))$  $+([mTORC1\_pS2448]*[Act\_ULK1]*0.00016)$  $- [Amino\_Acid]*[mTORC1]*par\_mTORC1\_S2448\_activation\_by\_Amino\_Acids$  $*( \frac{mTORC1_{vmax}*([leucine_{a}]+[leucine_{conc}])}{[leucine_{a}]+[leucine_{conc}]+[sestrin2]} )$  $-([Amino\_Acid]* [mTORC2]*par\_mTORC2\_S2481\_phos\_by\_Amino\_Acids)$  $-([PI3K\_p]*[mTORC2]*par\_mTORC2\_S2481\_phos\_by\_PI3K\_variant\_p)$  $+(par\_mTORC2\_pS2481\_dephos*[mTORC2\_pS2481])$  $+([Amino\_Acid]* [mTORC2]*par\_mTORC2\_S2481\_phos\_by\_Amino\_Acids)$  $+([PI3K\_p]*[mTORC2]*par\_mTORC2\_S2481\_phos\_by\_PI3K\_variant\_p)$  $-(par\_mTORC2\_pS2481\_dephos*[mTORC2\_pS2481])$  $-([Amino\_acid]*[IRS]*par\_IRS\_phos\_by\_Amino\_Acids)$  $-([IRS]* par\_IRS\_phos\_by\_IR\_beta\_pY1146*[IR\beta-pY1146])$  $-([IRS]*par\_IRS\_phos\_by\_p70\_S6K\_pT229\_pT389*$  $[p70\_S6K\_pT229\_pT389])$  $+(par\_IRS\_pS636\_turnover* [IRS\_pS636])$  $+([Amino\_Acid]* [IRS]* par\_IRS\_phos\_by\_Amino\_Acids)$  $+([IRS]* par\_IRS\_phos\_by\_IR\_beta\_pY1146* [IR\beta-pY1146])$  $-([IRS\_p]* par\_IRS\_p\_phos\_by\_p70\_S6K\_pT229\_pT389* [p70S6K\_pT229\_pT389])$  $+([IRS\_p]*par\_IRS\_p\_phos\_by\_p70\_S6K\_pT229\_pT389*[p70S6K\_pT229\_pT389])$  $+([IRS]* par\_IRS\_phos\_by\_p70\_S6K\_pT229\_pT389* [p70S6K\_pT229\_pT389])$  $-(par\_IRS\_pS636\_turnover*[IR\_pS636])$  $-([AMPK\_pT172]* [TSC1\_TSC2]*par\_TSC1\_TSC2\_S1387\_phos\_by\_AMPK\_pT172)$  $-(([AKT\_pT308]+[AKT\_pT3089\_pS473])*[TSC1\_TSC2]*par\_TSC1\_TSC2\_T1462\_phos\_by\_Akt\_pT308)$  $+(par\_TSC1\_TSC2\_pS1387\_dephos*[TSC1\_TSC2\_pS1387])$  $+(par\_TSC1\_TSC2\_pT1462\_dephos*[TSC1\_TSC2\_pT1462])$  $+(([Akt-pT308] +[Akt\_pT308\_pS473])*[TSC1\_TSC2]* par\_TSC1\_TSC2\_T1462\_phos\_by\_Akt\_pT308)$  $-(par\_TSC1\_TSC2\_pT1462\_dephos*[TSC1\_TSC2\_pT1462])$  $+([AMPK-pT172]* [TSC1\_TSC2]*par\_TSC1\_TSC2\_S1387\_phos\_by\_AMPK\_pT172)$  $-(par\_TSC1\_TSC2\_pS1387\_dephos*[TSC1\_TSC2\_pS1387])$  $-([PRAS40]*par\_PRAS40\_S183\_phos\_by\_mTORC1\_pS2448\_first*[mTORC1\_pS2448])$  $-(([Akt\_pT308]+ [Akt\_pT308\_pS473])*[PRAS40]*par\_PRAS40\_T246\_phos\_by\_Akt\_pT308\_first)$  $+(par\_PRAS40\_pS183\_dephos\_first*[PRAS40\_pS183])$  $+(par\_PRAS40\_pT246\_dephos\_first*[PRAS40\_pT246])$  $+([PRAS40]*par\_PRAS40\_S183\_phos\_by\_mTORC1\_pS2448\_first *[mTORC1\_pS2448])$  $-(([Akt\_pT308]+ [Akt\_pT308\_pS473])*[PRAS40\_pS183]*par\_PRAS40\_T246\_phos\_by\_Akt\_pT308\_second)$  $-(par\_PRAS40\_pS183\_dephos\_first*[PRAS40\_pS183])$  $+(par\_PRAS40\_pT246\_dephos\_second*[PRAS40\_pT246\_pS183])$  $-(par\_PRAS40\_S183\_phos\_by\_mTORC1\_pS2448\_second*[PRAS40\_pT246]* [mTORC1\_pS2448])$  $-(par\_PRAS40\_pT246\_dephos\_first*[PRAS40\_pT246])$  $+([Akt\_pT308]* [Akt\_pT308\_pS473]* [PRAS40]*par\_PRAS40\_T246\_phos\_by\_Akt\_pT308\_first)$  $+(par\_PRAS40\_pS183\_dephos\_second*[PRAS40\_pT246\_pS183])$  $+(([Akt\_pT308]+ [Akt\_pT308\_pS473])*[PRAS40\_pS183]*par\_PRAS40\_T246\_phos\_by\_Akt\_pT308\_second)$  $+(par\_PRAS40\_S183\_phos\_by\_mTORC1\_pS2448\_second* [PRAS40\_pT246]* [mTORC1\_pS2448])$  $-(par\_PRAS40\_pS183\_dephos\_second*[PRAS40\_pT246\_pS183])$  $-(par\_PRAS40\_pT246\_dephos\_second*[PRAS40\_pT246\_pS183])$  $-([AKT]* par\_Akt\_T308\_phos\_by\_PI3K\_p\_PDK1\_first* [PDK1\_p])$  $-([AKT]*par\_Akt\_S473\_phos\_by\_mTORC2\_pS2481\_first* [mTORC2\_pS2481])$  $+(par\_Akt\_pT308\_dephos\_first*[Akt\_pT308])$  $+(par\_Akt\_pS473\_dephos\_first*[Akt\_pS473])$  $+(par\_Akt\_T308\_phos\_by\_PI3K\_p\_PDK1\_second*[Akt\_pS473]* [PDK1\_p])$  $+(par\_Akt\_S473\_phos\_by\_mTORC2\_pS2481\_second*[Akt\_pT308]*[mTORC2\_pS2481])$  $-(par\_Akt\_pT308\_dephos\_first*[Akt\_pT308])$  $-(par\_Akt\_pS473\_dephos\_second*[Akt\_pT308\_pS473])$  $+([AKT]* par\_Akt\_T308\_phos\_by\_PI3K\_p\_PDK1\_first*[PDK1\_p])$  $-(par\_Akt\_S473\_phos\_by\_mTORC2\_pS2481\_second* [Akt\_pT308]* [mTORC2\_pS2481])$  $-(par\_Akt\_pT308\_dephos\_first*[Akt\_pT308])$  $+(par\_Akt\_pS473\_dephos\_second*[Akt\_pT308\_pS473])$  $+(par\_Akt\_pS473\_dephos\_second*[AKT\_pT308\_pS473])$  $-(par\_Akt\_pT308\_dephos\_first*[AKT\_pT308])$  $+(par\_PI3K\_PDK1\_phos\_by\_IRS\_p*[AKT]*[PI3K\_p\_PDK1])$  $-(par\_Akt\_S473\_phos\_by\_mTORC2\_pS2481\_first)*[AKT\_pT308]*[mTORC2\_pS2481])$  $-([PDK1\_p]* [p70S6K]*par\_p70\_S6K\_T229\_phos\_by\_PI3K\_p\_PDK1\_first)$  $-([mTORC1\_pS2448]* [p70S6K]* par\_p70\_S6K\_T389\_phos\_by\_mTORC1\_pS2448\_first)$  $+(par\_p70\_S6K\_pT229\_dephos\_first*[p70S6K\_pT229])$  $+(par\_p70\_S6K\_pT389\_dephos\_first*[p70S6K\_pT389])$  $+([PDK1\_p]*[p70S6K]*par\_p70\_S6K\_T229\_phos\_by\_PI3K\_p\_PDK1\_first)$  $-([mTORC1\_pS2448]*par\_p70\_S6K\_T389\_phos\_by\_mTORC1\_pS2448\_second*[p70S6K\_pT229])$  $+(par\_p70\_S6K\_pT389\_dephos\_second*[p70S6K\_pT229\_pT389])$  $-(par\_p70\_S6K\_pT229\_dephos\_first*[p70S6K\_pT229])$  $+([mTORC1\_pS2448]* [p70S6K]* par\_p70\_S6K\_T389\_phos\_by\_mTORC1\_pS2448\_first)$  $-([PDK1\_p]* par\_p70\_S6K\_T229\_phos\_by\_PI3K\_p\_PDK1\_second* [p70S6K\_pT389])$  $-(par\_p70\_S6K\_pT389\_dephos\_first*[p70S6K\_pT389])$  $+(par\_p70\_S6K\_pT229\_dephos\_second*[p70S6K\_pT229\_pT389])$  $+(\left[ PDK1_{p} \right]*par\_p70\_S6K\_T229\_phos\_by\_PI3K\_p\_PDK1\_second* [p70S6K\_pT389])$  $+(\left[ mTORC1_{pS2448} \right]*par\_p70\_S6K\_T389\_phos\_by\_mTORC1\_pS2448\_second*[p70S6K\_pT229])$  $-(par\_p70\_S6K\_pT229\_dephos\_second*[p70S6K\_pT229\_pT389])$  $-(par\_p70\_S6K\_pT389\_dephos\_second*[p70S6K\_pT229\_pT389])$  $+(par\_PI3K\_variant\_p\_dephos*[PI3K\_p])$  $-([IR\beta\_pY1146]*[PI3K]*par\_PI3K\_variant\_phos\_by\_IR\_beta\_pY1146)$  $-(par\_PI3K\_variant\_p\_dephos*[PI3K\_p])$  $+([IR\beta\_pY1146]*[PI3K]*par\_PI3K\_variant\_phos\_by\_IR\_beta\_pY1146)$  $+(par\_PI3K\_p\_PDK1\_dephos*[PDK1\_p])$  $-([IRS\_p]* [PDK1]* par\_PI3K\_PDK1\_phos\_by\_IRS\_p)$  $-(par\_PI3K\_p\_PDK1\_dephos*[PDK1\_p])$  $+([IRS\_p]* [PDK1]* par\_PI3K\_PDK1\_phos\_by\_IRS\_p)$  $-(100*[AMPK]*par\_AMPK\_T172\_phos\_by\_AminoAcids*[leucine\_conc]*[sestrine\_conc])$  $-([AMPK]* par\_AMPK\_T172\_phos* [IRS\_p])$  $+(par\_AMPK\_pT172\_dephos*[AMPK\_pT172])$  $-(ksirt*[SIRT]*[AMPK])$  $+([Act\_ULK1]*[AMPK\_pT172]*kUKAP)$  $+(100*[AMPK]*par\_AMPK\_T172\_phos\_by\_AminoAcids*[leucine\_conc]*[sestrine\_conc])$  $+([AMPK]* par\_AMPK\_T172\_phos* [IRS\_p])$  $-(par\_AMPK\_pT172\_dephos*[AMPK\_pT172])$  $+(ksirt*[SIRT]*[AMPK])$  $-([Act\_ULK1]*[AMPK\_pT172]*kUKAP)$  $+kAKT*([Akt\_pT308]+[Akt\_pT308\_pS473])*[FOXO\_AC\_P] - kAMPK*[AMPK\_pT172]*[FOXO\_de]$  $+ kAMPK*[FOXO\_de]*[AMPK\_pT172] - kAKT*([Akt\_pT308\_pS473]+[Akt\_pT308])*[FOXO\_AC\_P]$  $- kSIRTFOXO*\left[ FOXO\_AC\_P \right]*[SIRT] + kCBp*[FOXO\_P]*[CBP]$  $kSIRTFOXO*[FOXO\_AC\_P]*[SIRT] - kCBp*[FOXO\_P]*[CBP]$  $+ kdn*[ AC\_PGC1\alpha]$  $-\left( \frac{Vspgc* PGC1\alpha*SIRT}{kAPGC+SIRT} \right)$  +$Vopgc$  $-\left( \frac{Vspgc* PGC1\alpha*SIRT}{kAPGC+SIRT} \right)$  $-\left( Vdpgc*\frac{\left[ AC\_PGC1\alpha\right]}{kdpgc+ \left[ AC\_PGC1\alpha\right]} \right)$  $- kdn* [AC\_PGC1\alpha]$  $-k10*[ULK1]*[AMPK\_pT172] + kULKd*[Act\_ULK1] + KULKM*[Act\_ULK1]*[mTORC1\_pS2448]$  $k10*[ULK1]*[AMPK\_pT172] - kULKd*[Act\_ULK1] - KULKM*[Act\_ULK1]*[mTORC1\_pS2448]$  $(-(NAMimport) + 0.9625*(NAMimport) + (Nam\_uptake))$  $(1/NamPT\_compartment * (NAD\_binding))$  $(1/NamPT\_compartment * ((NT5\_NMN) - (PNP\_NR) - (NR\_efflux) - (NRK1\_NMN)))$  $(1/NamPT\_compartment * ((NMNAT1\_NaMN) - (NADS)))$    $(1/NamPT\_compartment * (-(NMN\_efflux) - (NMNAT1\_NMN) - (NT5\_NMN) + (NAMPT) + (NRK1\_NMN)))$  $(1/NamPT\_compartment * (-(NAPRT) + (NADA) - (NA\_efflux) + (PNP\_NAR)))$  $(1/NamPT\_compartment * ((NAPRT) - (NMNAT1\_NaMN) - (NT5\_NaMN) + (NRK1\_NaMN)))$  $(1/NamPT\_compartment * ((PNP\_NR) - (NNMT) - (Nam\_efflux) + (SIRT) +$  $(NAD\_consumption\_without\_Nam\_inhibition) - (NADA) + (NAMimport) - (NAMPT)))$  $(1/NamPT\_compartment * ((NMNAT1\_NMN) + (NADS) - (SIRT) - (NAD\_consumption\_without\_Nam\_inhibition)$  $- (NAD\_binding) - (NAD\_efflux)))$  $(1/NamPT\_compartment * ((NT5\_NaMN) - (NAR\_efflux) - (NRK1\_NaMN) - (PNP\_NAR)))$  $(NamPT\_compartment * celldivision\_rate\_NamPT\_compartment * NMN)$    $\left( \frac{NamPT\_ compartment*NAPRT\_E\_T* NAPRT\_turnovver* NA * ATP*NAPRT\_scaling}{NAPRT\_Km+ NA} \right)$    $\frac{\begin{aligned} NamPT\_compartment * (ETNMNAT * NMNAT1\_NMN\_scaling * \\ \left( \left( NMNAT\_NMN\_kcat\_A*\frac{NMN}{NMNAT\_NMN\_km\_A} \right)-\left( NMNAT1\_NMN\_kcat\_PA*\frac{NAD}{NMNAT1\_NMN\_km\_PA} \right) \right) \end{aligned}}{1+\frac{NMN}{NMNAT1\_NMN\_Km\_A}+\frac{NaMN}{NMNAT1\_NMN\_Km\_B}+\frac{NAD}{NMNAT1\_NMN\_Km\_PA}+\frac{NaAD}{NMNAT1\_NMN\_Km\_PB}}$  $\frac{NamPT\_compartment * (ETNMNAT * NMNAT1\_NaMN\_scaling * \left( \frac{NMNAT1\_NaMN\_kcat\_A*NaMN}{NMNAT1\_NaMN\_Km\_A} - \frac{NMNAT1\_NAMN\_kcat\_PA*NaAD}{NMNAT1\_NaMN\_Km\_PA} \right)}{1+\frac{NaMN}{NMNAT1\_NaMN\_Km\_A}+\frac{NMN}{NMNAT1\_NaMN\_Km\_B}+\frac{NaAD}{NMNAT1\_NaMN\_Km\_PA}+\frac{NAD}{NMNAT1\_NaMN\_Km\_PB}}$    $\frac{NamPT\_compartment *NADS\_E\_T * NADS\_turnover * NaAD * ATP *NADS\_scaling}{(NADS\_Km + NaAD}$    $\frac{NamPT\_compartment * NT5\_NMN\_ET * NT5\_NMN\_scaling * NT5\_NMN\_kcat*NMN}{NT5\_NMN\_Km + NMN}$    $\frac{NamPT\_compartment *PNP\_NR\_E\_T * PNP\_NR\_turnover * NR * Pi *PNP\_NR\_scaling}{PNP\_NR\_Km + NR}$    $\frac{NamPT\_compartment* NNMT\_ET* NNMT\_scaling* NNMT\_Kcat* SAM*NAM}{Kma * (Kmb + NAM)* (1 +\frac{methyl\_NAM}{NNMTki})+ SAM*Kmb + SAM*NAM}$    $(NamPT\_compartment * celldivision\_rate\_NamPT\_compartment * NAM)$    $\frac{NamPT\_compartment * NT5\_NaMN\_ET * NT5\_NaMN\_scaling * NT5\_NaMN\_kcat*NaMN}{NT5\_NaMN\_Km + NaMN}$    $\frac{NamPT\_compartment * SIRT\_ET * SIRT\_scaling * SIRT_{Kcat}* NAD *H3_{ac}}{((SIRT\_Km + NAD) * (1+\frac{NAM}{SIRT\_Ki}))}$    $NamPT\_compartment*NAD\_consumption\_without\_Nam\_inhibition\_ET**\frac{NAD\_consumption\_without\_Nam\_inhibition\_scaling*NAD\_consumption\_without\_Nam\_inhibition\_kcat *NAD}{NAD\_consumption\_without\_Nam\_inhibition\_Km+NAD}$  $\frac{NamPT\_compartment*NADA\_ET*NADA\_scaling*NADA_{Kcat}*NAM}{NADA_{Km}+NAM+\frac{NADA\_Km*NA}{NADA\_Ki}}$    $(NamPT\_compartment*(Nam\_transporter*Nam\_ex-Nam\_transporter*NAM))$      $\frac{NamPT\_compartment*((NAMPT\_ET*(NAMPT\_scaling*\left( Kcat\_Namprt \right)*NAM*\left( 0.8+0.2*\frac{pAMPK}{15} \right)}{(Km\_NamPRT)+NAM+(Km\_NamPRT)*\frac{NAD}{NAMPT\_Ki)}}$    $NamPT\_compartment*celldivision\_rate\_NamPT\_compartment*NAR$    $NamPT\_compartment*celldivision\_rate\_NamPT\_compartment*NA$    $NamPT\_compartment*(NAD\_binding\_k1*NAD-NAD\_binding\_k2*NADbound)$    $\frac{NamPT\_compartment*NRK1\_NaMN\_ET*NRK1\_NaMN\_scaling*NRK1\_NaMN\_kcat*NAR}{NRK1\_NaMN\_Km+NAR}$    $\frac{NamPT\_compartment*PNP\_NAR\_E\_T*PNP\_NAR\_turnover*NAR*Pi*PNP\_NAR\_scaling}{PNP\_NAR\_Km+NAR}$  $NamPT\_compartment*celldivision\_rate\_NamPT\_compartment*NR$    $\frac{NamPT\_compartment*NRK1\_NMN\_ET*NRK1\_NMN\_kcat*NRK1\_NMN\_scaling*NR}{\left( NRK1\_NMN\_Km+NR \right)}$ |
| --- | --- |


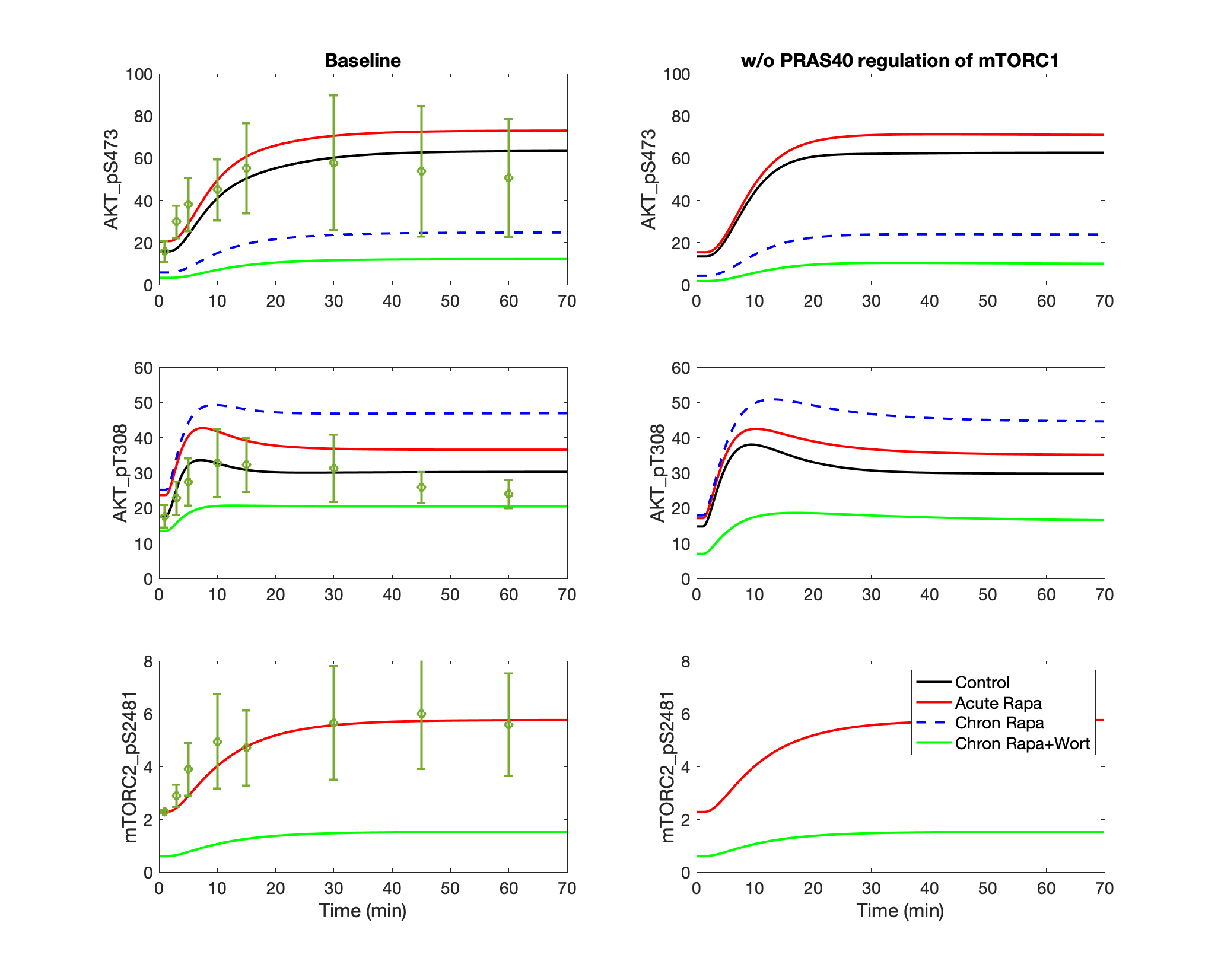


**Figure S2.** Effects of insulin, rapamycin, and wortmannin on key proteins and their interactions. Insulin was lowered to 10% of its baseline level for the initial 40 min of the simulation, and subsequently returned to baseline level. Simulations are conducted for control, acute and chronic administration of rapamycin, chronic administration of rapamycin with wortmannin. The inhibition of PRAS40 of mTORC1 is represented in the left panels but not the right ones. Included also are experimental time-course data from Dalle Pezze et al. (2016); points and dotted error bars represent mean and SEM.


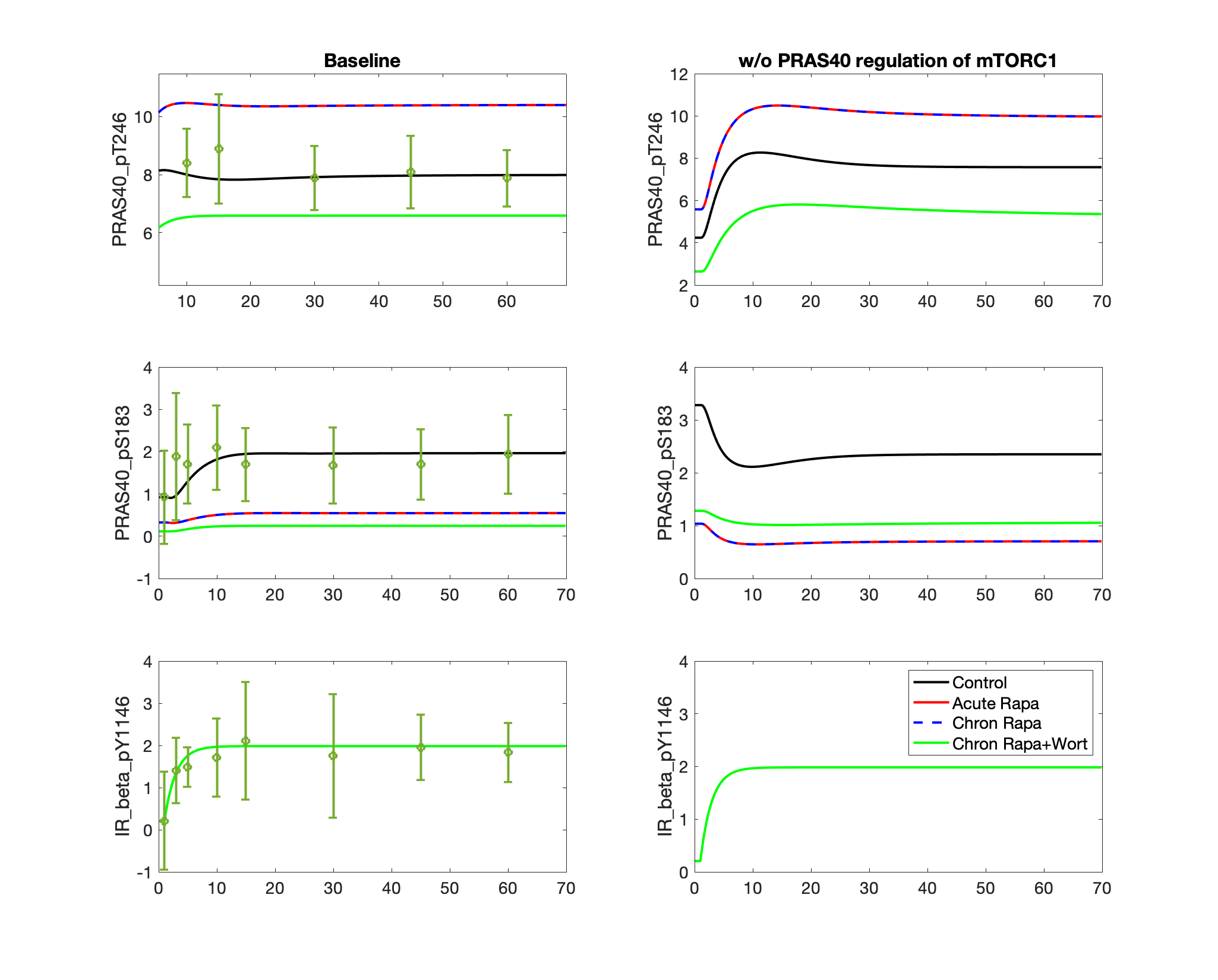


**Figure S3.** Effects of insulin, rapamycin, and wortmannin on key proteins and their interactions. Notations are analogous to Fig. S2.


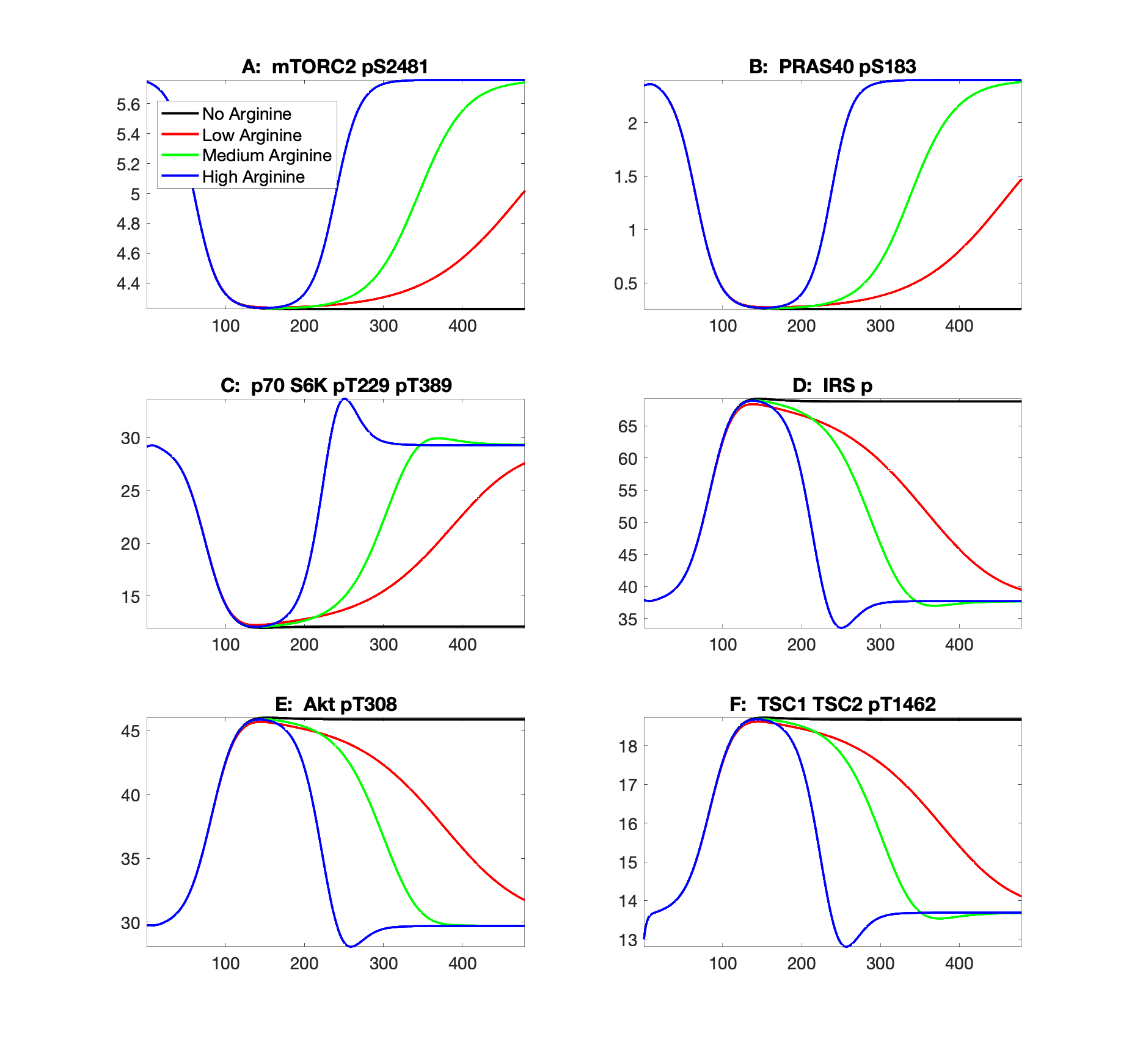


**Figure S4.** Effect of protein deprivation and subsequent leucine efflux on key model variables, obtained for differing arginine levels.


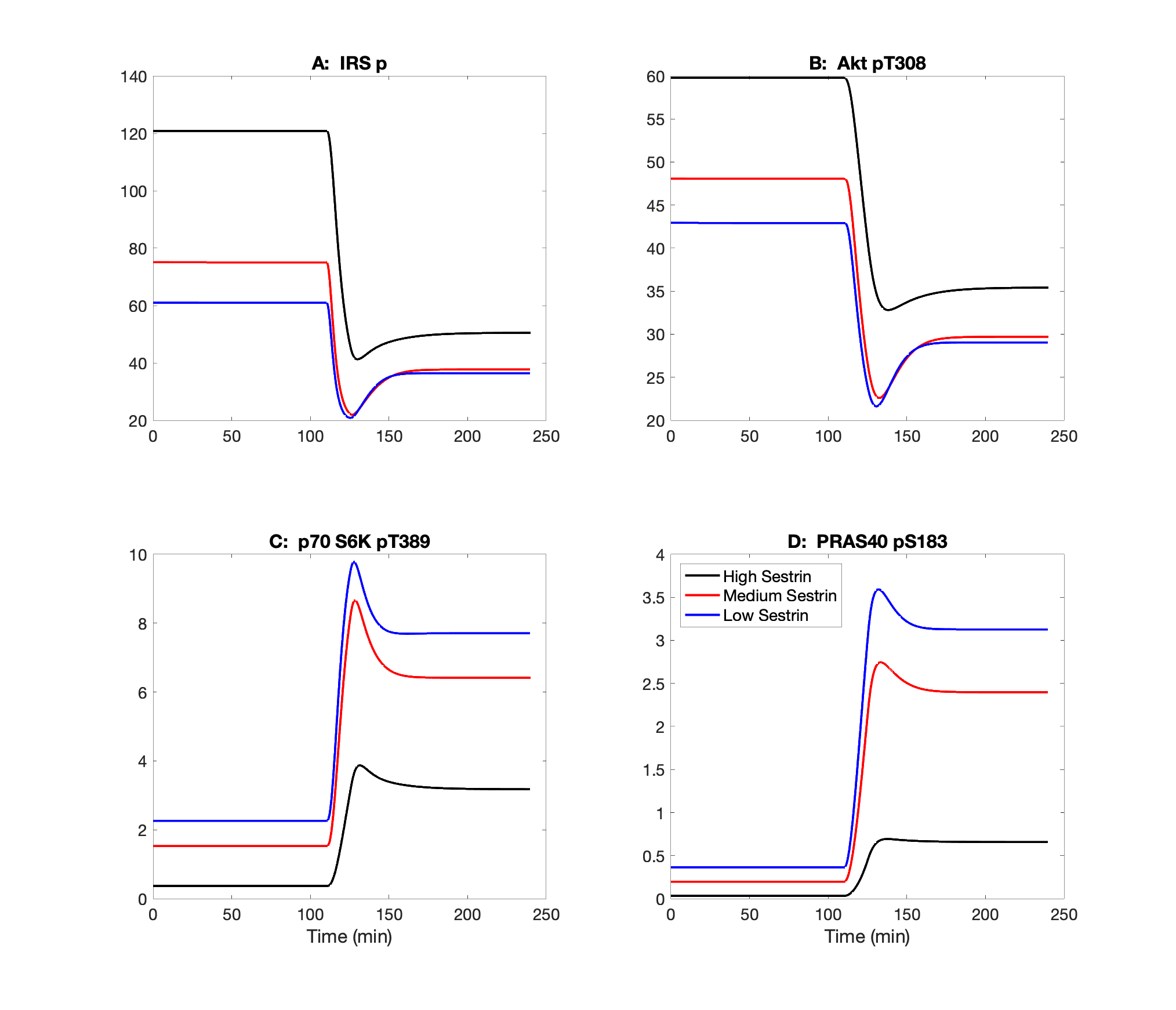
 **Figure S5.** Effect of protein depletion and restoration on key model variables, obtained for differing sestrin2 levels.
